# A novel protein encoded by circUBE4B promotes progression of esophageal squamous cell carcinoma by augmenting MAPK/ERK signaling

**DOI:** 10.1038/s41419-023-05865-2

**Published:** 2023-06-01

**Authors:** Yingcheng Lyu, Binghua Tan, Lin Li, Ruihao Liang, Kai Lei, Kefeng Wang, Duoguang Wu, Huayue Lin, Minghui Wang

**Affiliations:** 1 Guangdong Provincial Key Laboratory of Malignant Tumor Epigenetics and Gene Regulation, Sun Yat-sen Memorial Hospital, Sun Yat-sen University, 510120 Guangzhou, China; 2 Department of Thoracic Surgery, Sun Yat-sen Memorial Hospital, Sun Yat-sen University, 510120 Guangzhou, China; 3 Breast Tumor Center, Sun Yat-sen Memorial Hospital, Sun Yat-sen University, 510120 Guangzhou, China

**Keywords:** Cancer, Tumour biomarkers

## Abstract

Esophageal squamous carcinoma (ESCC) is a common malignant cancer. Although the non-coding roles of circRNAs in the pathogenesis of human tumors have been well studied, whether circRNAs participate in the progression of ESCC by encoding novel proteins remains unclear. In this study, we identified an overexpression circRNA with protein-coding ability in ESCC tissues, called circUBE4B, whose expression level is correlated with tumor size and tumor differentiation level of ESCC patients. Moreover, a higher level of circUBE4B in ESCC patients is correlated with a worse prognosis. Functionally, we found that circUBE4B promoted the proliferation of ESCC cells by encoding a novel cancer-promoting protein, circUBE4B-173aa. Mechanistically, the circUBE4B-173aa protein interacts with MAPK1 and promotes the phosphorylation level of MAPK1 to eventually activate MAPK/ERK signaling pathway. The xenograft model revealed that overexpression of circUBE4B-173aa in ESCC cells significantly promoted the growth of grafts. Our study provides new insights into the mechanism of circRNA in the development of ESCC and circUBE4B-173aa has the potential to serve as a biomarker and a novel therapeutic target for ESCC therapy.

## Introduction

Esophageal cancer is a primary malignant tumor with high morbidity and mortality rates, characterized by aggressive growth and a high recurrence rate [1]. There are two main histopathological subtypes of esophageal cancer, including esophageal squamous carcinoma(ESCC) and esophageal adenocarcinoma(EAC), with ESCC accounting for approximately 90% of esophageal cancer cases worldwide [2]. In the past 20 years, although advances in clinical diagnosis and treatment have made great progression, the overall 5-year survival rate is less than 20% for patients with ESCC [3]. Therefore, it is urgent to explore the comprehensive molecular mechanisms involved in the occurrence and progression of esophageal cancer, and to search for the therapeutic targets and effective prognostic markers to further improve the survival time of esophageal cancer patients.

With the development of RNA high-throughput sequencing technology and advances in biotechnology, it has been discovered that numerous circRNAs perform multiple biological functions in the development of cancer [4]. Previous studies have suggested that circRNAs are a variety of classical non-coding RNAs [5] that are formed by pre-miRNAs by reverse splicing the covalently linked 3′-end and 5′-end. CircRNAs perform their biological functions mainly by acting as microRNA (miRNA) sponges [6], regulating gene transcription [5, 7], adsorbing RNA-binding proteins [8], and regulating protein translation [9]. Recently, a previously hidden role for circRNAs was highlighted, that circRNAs with internal ribosomal entry sites (IRES) and open reading frames (ORF) have the capacity to encode proteins [10–15].

We reported previously that circGSK3β promoted ESCC cell migration and invasion via direct interaction with GSK3β and inhibiting GSK3β activity [16], while hsa_circ_0006948 enhances ESCC progression and epithelial-mesenchymal transition through the miR-490-3p sponging [17], indicating that circRNAs are involved in ESCC progression and metastasis. However, the understanding of circRNAs with translation potency and the functional and clinical significance of circRNA-encoded proteins (circ-proteins) in ESCC remains to be further explored.

In this study, we showed that circUBE4B promoted ESCC proliferation by encoding a novel protein, circUBE4B-173aa and augmenting MAPK/ERK signaling. Furthermore, overexpression of circUBE4B and circUBE4B-173aa were associated with poor outcomes in human ESCC patients. Overexpression of circUBE4B in ESCC cells promoted tumorigenicity when they were grafted in mice. These results reveal that aberrantly expressed circRNA can also affect biological progression by translating protein. They also uncover the potential of circUBE4B/circUBE4B-173aa as a therapeutic target for ESCC treatments and as a biomarker for ESCC prognosis.

## Results

### CircUBE4B is upregulated in ESCC and its high level predicts a poor prognosis

**Fig. 1:**
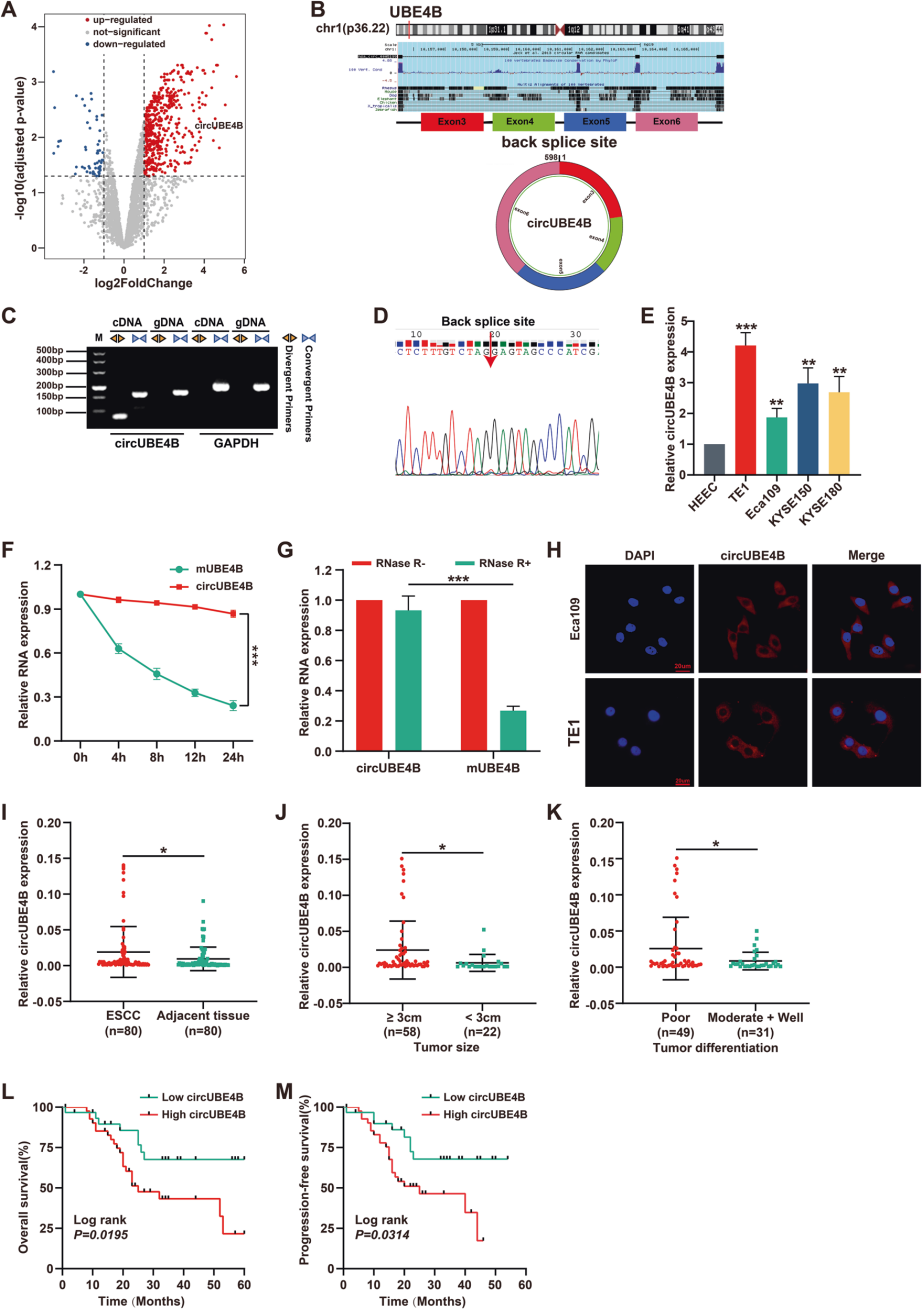
CircUBE4B is upregulated in ESCC and its high level predicts poor prognosis. **A** Volcano plot showing circRNAs that are dysregulated in ESCC tissues compared to matched adjacent normal tissues. Red dots indicate significantly upregulated circRNAs and blue dots indicate significantly downregulated circRNAs. CircUBE4B (hsa_circ_0005199) is significantly upregulated in ESCC tissues. **B** Diagram showing that circUBE4B originates from back splicing of exons 3 and 6 of the linear form of UBE4B mRNA. **C** Amplification of circUBE4B from cDNA and gDNA using convergent primers and divergent primers, respectively. **D** Sanger sequencing confirmed the reverse splice site of circUBE4B. **E** The relative expression levels of circUBE4B in different ESCC cells were detected by qRT-PCR. **F** qRT-PCR analysis of the relative levels of circUBE4B and linear form of UBE4B mRNA in TE1 cells treated with actinomycin D (10 μg/ml). **G** The relative expression levels of circUBE4B and linear form of UBE4B mRNA were detected by qRT-PCR after treating total RNA from TE1 cells with RNase R. **H** Subcellular localizations of circUBE4B in TE1 and Eca109 cells were detected by RNA-FISH (bar = 20 μm). **I** Graphs showing the results of the relative expressions of circUBE4B detected by qRT-PCR in 80 pairs of ESCC tissues compared to adjacent normal tissues. **J**, **K** Statistical analysis of circUBE4B expressions in different tumor size (**J**) and tumor differentiation (**K**) samples. **L** Kaplan–Meier analysis of the correlation between circUBE4B expression and overall survival (OS) in ESCC patients. **M** Kaplan–Meier analysis of the correlation between circUBE4B expression and progression-free survival (PFS) in ESCC patients. *P < 0.05, **P < 0.01, ***P < 0.001.

To investigate the circRNAs with translational functions in ESCC, we compared the circRNA expression profiles of three pairs of ESCC tissues and their matched adjacent normal tissues from the GEO dataset (GSE131969). Based on the baseline of log_2_ (fold change) ≥1, *p* < 0.05, the differential expressions of circRNA in esophageal cancer and adjacent normal tissues are shown in the volcano diagram (Fig. 1A). We searched for those with open reading frame (ORF) and a segment of the internal ribosome entry site (IRES) from the differentially expressed circRNA and hsa_circ_0005199 emerged as the most upregulated one with protein-coding potential in ESCC tissues than paired adjacent normal tissues (Table S1). Hsa_circ_0005199 is formed by reverse splicing of exons 6–9 of the linear transcript of the ubiquitination factor E4B (UBE4B) gene with a length of 598 nucleotides, which we call circUBE4B (Fig. 1B). Then, we designed and synthesized two sets of primers. Convergent primers are only used to amplify the linear structure of UBE4B, the mRNA, while divergent primers are only used to amplify the circular structure, the circUBE4B. Using complementary DNA (cDNA) and genomic DNA (gDNA) extracted from Eca109 and TE1 cells as templates, nucleic acid gel electrophoresis showed that circUBE4B was amplified only from cDNA but not from gDNA (Fig. 1C), indicating that the circular structure of circUBE4B was generated by reverse splicing. To further determine the loop structure of circUBE4B, we confirmed its reverse splice site by Sanger sequencing (Fig. 1D). To further study whether circUBE4B was also overexpressed in ESCC cells, quantitative real-time PCR (qRT-PCR) was carried out to assess the expression of circUBE4B in ESCC cells TE1, Eca109, KYSE-150, and KYSE-180, as well as normal esophageal epithelial cell line HEEC. The results showed that expression of circUBE4B was higher in ESCC cells than in normal human esophageal epithelial cells (Fig. 1E). We then further evaluated the stability and subcellular localization of circUBE4B. After treating ESCC cells with actinomycin D, we found that the half-life of circUBE4B was significantly longer than that of the linear form of UBE4B mRNA (Fig. 1F). Contrast to the linear form of UBE4B mRNA, circUBE4B is resistant to RNase R degradation, which also indicates that circUBE4B is highly stable in ESCC cells (Fig. 1G). Subsequently, using fluorescence in situ hybridization (FISH) experiments, we found that circUBE4B was mainly localized at the cytoplasm (Fig. 1H).

To verify the overexpression of circUBE4B in ESCC, qRT-PCR was carried out to assess the expression of circUBE4B in a total amount of 80 ESCC tissues and paired adjacent non-cancerous tissues. Consistent with our previous report in ESCC cells, circUBE4B was significantly upregulated in cancer tissues compared to its adjacent normal tissues (Fig. 1I). To further understand the significance of circUBE4B in ESCC, the correlation between circUBE4B expressions and clinicopathological characteristics were analyzed (Table 1). The median expression value of all 80 cases was chosen as the cutoff value for separating the dataset into a circUBE4B-low group and circUBE4B-high group. The results showed that circUBE4B expression levels were significantly associated with the tumor sizes (Fig. 1J) and differentiation (Fig. 1K) in ESCC (Table S2). In addition, to determine whether circUBE4B expression correlated with the prognosis of ESCC patients, the association of circUBE4B expression and the ESCC overall survival (OS) (Fig. 1L) and progression-free survival (PFS) (Fig. 1M) times of the 80 patients were analyzed. Kaplan–Meier analysis revealed a reverse association between the expression levels of circUBE4B and the OS time of the patients, as well as with the PFS time. Overall, our findings demonstrate that circUBE4B is upregulated in ESCC tissues and the level of circUBE4B expression has the potential to serve as a biomarker for predicting outcomes of ESCC patients.

**Table 1:** Relationship between clinicopathological characteristics and circUBE4B expression in ESCC patients.

Clinicopathological parameters	Number	circUBE4B expression level		
		High	Low	*P* value
**Sex**				
male	69	42	27	0.7473
Female	11	6	5	
**Age**		60.25 ± 8.21	62.88 ± 9.34	0.1890
**T stage**^**1**^				0.9197
T1 + T2	23	14	9	
T3 + T4	57	34	23	
**N stage**^**a**^				0.8200
N0	37	23	14	
N1 + N2 + N3	43	25	18	
**TNM stage**^**a**^				0.1565
I + II	27	12	15	
III-IV	53	33	20	
**Tumor size**				0.0421^*^
<3 cm	22	9	13	
≥3 cm	58	39	19	
**Differentiation**				0.0375^*^
Moderate + Well	31	14	17	
Poor	49	34	15	

^**a**^Tumor, node, metastasis stage (TNM stage).

*P < 0.05.

### CircUBE4B promotes the proliferation of ESCC cells

**Fig. 2:**
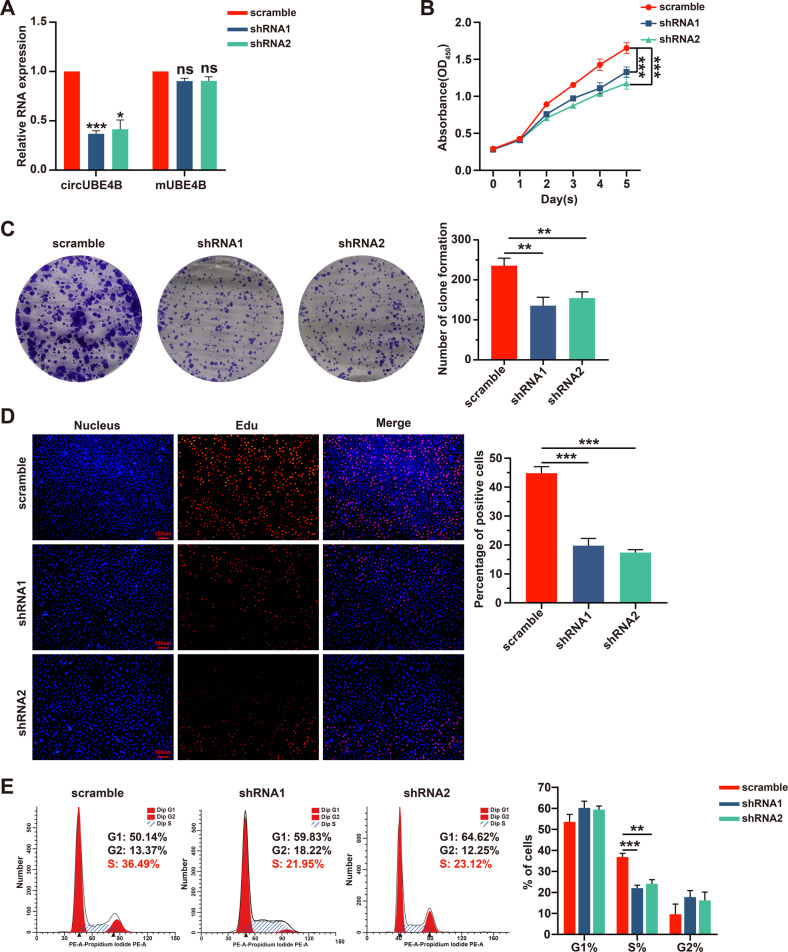
CircUBE4B promotes the proliferation of ESCC cells. **A** qRT-PCR analysis of circUBE4B and linear form of UBE4B mRNA in ESCC cells after circUBE4B silencing by circUBE4B specific shRNA plasmid. **B** The proliferation ability of ESCC cells were detected by CCK8 assays after treatments with circUBE4B shRNA plasmids. **C** The proliferation ability of ESCC cells were detected by clone formation assay after treatments with circUBE4B shRNA plasmids. **D** The proliferation ability of ESCC cells were detected by Edu assay after treatments with circUBE4B shRNA plasmids (bar = 100 μm). **E** Cell cycle changes in ESCC cells were assessed by flow cytometry after treatments with circUBE4B shRNA plasmids. *P < 0.05, **P < 0.01, ***P < 0.001.

To investigate the biological functions of circUBE4B in ESCC, circUBE4B was depleted stably by the expression of circUBE4B specific shRNAs, which targeted the back-splicing region. The qRT-PCR results showed that depletion of circUBE4B resulted in reducing the level of circUBE4B in TE1 cells, without affecting the mRNA expression level of UBE4B (Fig. 2A). To determine whether the expression level of circUBE4B affected cell proliferation, the CCK8 assay was carried out. As shown in Fig. 2B, the knockdown of circUBE4B significantly inhibited the proliferative capacity of ESCC cells. Consistently, clone formation assay (Fig. 2C) and Edu experiments (Fig. 2D) also confirmed the same results. Additionally, we detected whether circUBE4B could regulate the cell cycle of ESCC cells. Flow cytometry analysis showed that the knockdown of circUBE4B significantly reduced the proportion of ECSS cells in the S-phase (Fig. 2E). Taken together, the results demonstrate that circUBE4B contributed to the proliferation of ESCC cells.

### CircUBE4B encodes a novel protein, circUBE4B-173aa, in ESCC

**Fig. 3:**
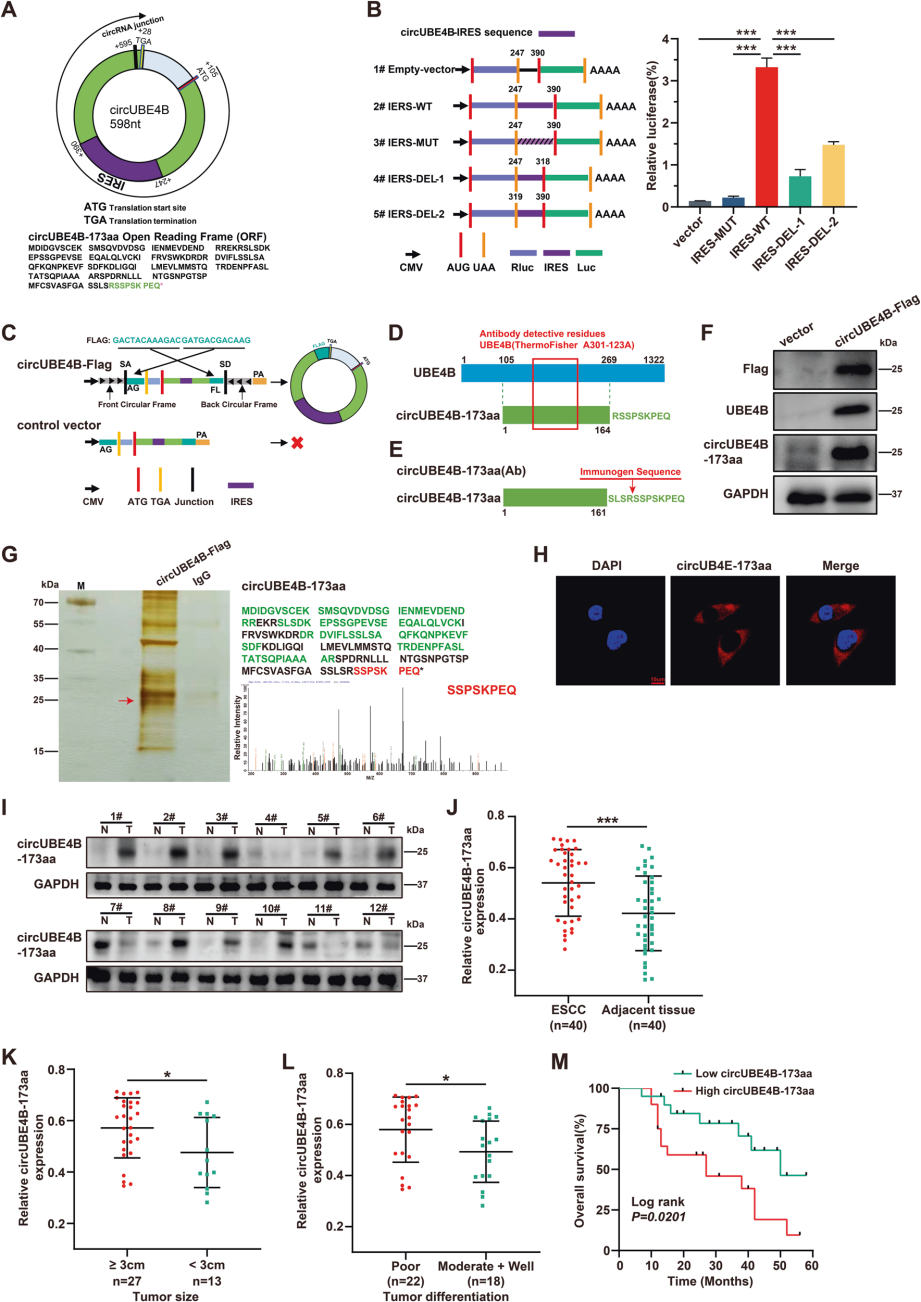
CircUBE4B encodes a novel protein, circUBE4B-173aa, in ESCC. **A** Above, schematic diagram of the predicted start codon (ATG), stop codon (TGA), and IRES sequence of ORF in circUBE4B. Below, the predicted amino acid sequence of circUBE4B-173aa, a novel protein encoded by circUBE4B. **B** Detection activity of IRES in circUBE4B. Left, circUBE4B wild-type sequence or its truncated, mutated IRES sequence was cloned between Rluc and Luc reporter genes with an independent start codon (AUG) and stop codon (UAA). Right, the relative luciferase activity of Luc/Rluc in each group were examined. **C** Illustration of the synthetic circRNA overexpression plasmids: circUBE4B-Flag, the full-length sequence of circUBE4B were cloned between SA and SD with both sides of flanking repeat sequences as indicated, with triple Flag tag sequences were separately cloned at both sides; control vector, SA, SD, and flanking repeat sequences were removed from circUBE4B-Flag vector to prevent circularization. **D** The UBE4B (A301-123A) antibody used in the study recognizes the novel protein encoded by circUBE4B and its parental protein UBE4B. **E** A circUBE4B-173aa antibody was generated to target these N-terminal unique sequences. **F** The total proteins of Eca109 cells after transfection with control plasmids and circUBE4B-Flag plasmids were obtained, and Western Blot confirmed the overexpression of Flag and circUBE4B-173aa. **G** Left, after transfection of circUBE4B-Flag or control plasmids into Eca109 cells, total proteins from transfected Eca109 cells were separated by SDS-PAGE and the gel band near 25 kDa was cut down for liquid chromatography-tandem mass spectrometry (LC-MS/MS) analysis. Right, the amino acid sequences identified by mass spectrometry was marked in green, and the specific peptide sequence SSPSKPEQ of the circUBE4B-173aa was marked in red. **H** The subcellular localization of circUBE4B-173aa was detected by immunofluorescence staining using anti-Flag (bar = 10 μm). **I** CircUBE4B-173aa expression levels were detected in ESCC tissues and adjacent normal tissues. **J** Graphs showing the results of the relative expressions of circUBE4B-173aa detected by Western Blot in 40 pairs of ESCC tissues compared to adjacent normal tissues. **K**, **L** Statistical analysis of circUBE4B-173aa expressions in different tumor size (**K**) and tumor differentiation (**L**) samples. **M** Kaplan–Meier analysis of the correlation between circUBE4B-173aa expression and OS in ESCC patients. *P < 0.05, ***P < 0.001.

To gain insight into the molecular mechanism by which circUBE4B contributed to the proliferation of ESCC, we further validated the encoding potential of circUBE4B. According to predictions from the online databases circRNADb [18] and TransCirc [19], the sequence of circUBE4B contains an ORF and a segment of the IRES located at 247-390nt. This result suggests that circUBE4B has the potential to encode a new protein of length 173aa, which we named circUBE4B-173aa in this study (Fig. 3A). Dual-luciferase reporter assay was explored to examine the putative IRES activity in circUBE4B. The results showed that the luciferase activity of the wild-type IRES reporter gene was significantly higher than that of the mutant and truncated IRES reporter gene (Fig. 3B), indicating that IRES of circUBE4B might be useful in initiating translation. To validate the expression of circUBE4B-173aa, circUBE4B with triple Flag tag was overexpressed in ESCC cells (Fig. 3C). The Western Blot results demonstrated that Flag antibody, UBE4B antibody (A301-123A) (Fig. 3D) and circUBE4B-173aa specific antibody (Fig. 3E) detected the significant protein bands at approximately 25 kDa (Fig. 3F and Fig. S1A), which was exactly at the predicted size of circUBE4B-173aa. What’s more, we performed immunoprecipitation experiments (IP), followed by mass spectrometry to confirm the sequence of circUBE4B-173aa. Indeed, a major band at 25 kDa was identified to be circUBE4B-173aa, which contains the specific sequence of the back-splicing region (SSPSKPEQ) (Fig. 3G and Fig. S1B). Next, we determined the subcellular localization of circUBE4B-173aa in ESCC cells by immunofluorescence (IF) assays and the results showed that circUBE4B-173aa was mainly located in the cytoplasm (Fig. 3H).

To investigate the clinical significance of circUBE4B-173aa in ESCC, we further assessed the expression of circUBE4B-173aa in ESCC tissues and adjacent normal tissues. By using Western Blot, we validated that the expression of circUBE4B-173aa was remarkably overexpressed in ESCC compared with adjacent normal tissues (Fig. 3I, J and Fig. S1C-E). The correlation between circUBE4B-173aa expressions and clinicopathological characteristics was analyzed (Table 2). Further analysis showed that the expression level of circUBE4B-173aa significantly correlated with the tumor size (Fig. 3K) and differentiation (Fig. 3L) in ESCC patients (Table S3). Kaplan–Meier analysis showed that ESCC patients with higher expression of circUBE4B-173aa had significantly shorter overall survival (OS) than those with lower circUBE4B-173aa expression (Fig. 3M). Altogether, we identified a novel protein, circUBE4B-173aa, encoded by circUBE4B in ESCC cells. What’s more, higher levels of circUB4E4B-173aa in ESCC were associated with poor outcomes.

**Table 2:** Relationship between clinicopathological characteristics and circUBE4B-173aa expression in ESCC patients.

Clinicopathological parameters	Number	circUBE4B-173aa expression level		
		High	Low	*P* value
**Sex**				
male	23	13	10	0.7471
Female	17	11	6	
**Age**		59.80 ± 9.431	64.05 ± 8.918	0.1609
**T stage**^**a**^				0.1018
T1 + T2	19	9	10	
T3 + T4	21	16	5	
**N stage**^**a**^				0.1748
N0	15	8	7	
N1 + N2 + N3	25	19	6	
**TNM stage**^**a**^				0.0939
I + II	16	7	9	
III-IV	24	18	6	
**Tumor size**				0.0312^*^
<3 cm	13	5	8	
≥3 cm	27	21	6	
**Differentiation**				0.0230^*^
Moderate + Well	18	7	11	
Poor	22	17	5	

^**a**^Tumor, node, metastasis stage (TNM stage).

*P < 0.05

### CircUBE4B promoted the proliferation of ESCC cells by its encoded protein circUBE4B-173aa

**Fig. 4:**
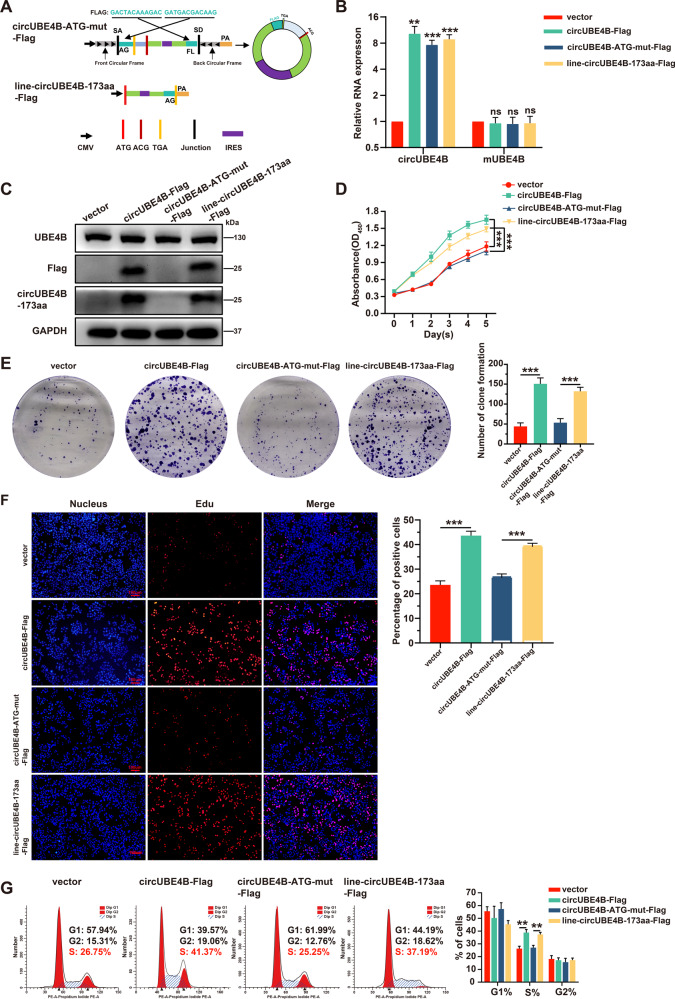
CircUBE4B promoted the proliferation of ESCC cells by its encoded protein circUBE4B-173aa. **A** Illustration of the other two synthetic circRNA expression plasmids: circUBE4B-ATG-mut-Flag, the full-length sequence of circUBE4B with a mutated start codon (ATG → ACG) were cloned between SA and SD with both sides of flanking repeat sequences as indicated, with triple Flag tag sequences were separately cloned at both sides, Line-circUBE4B-173aa-Flag, the open reading frames sequence of circUBE4B with triple Flag tag sequences were cloned into overexpressing plasmids as indicated, SA, SD, and flanking repeat sequences were removed to prevent the linear sequence from cyclization. **B** qRT-PCR analysis of circUBE4B and linear form of UBE4B mRNA in ESCC cells after treatments with control plasmids, circUBE4B-Flag plasmids, circUBE4B-ATG-mut-Flag plasmids, and line-circUBE4B-173aa-Flag plasmids. **C** The expressions of Flag, circUBE4B-173aa, and UBE4B in ESCC cells were detected by Western Blot after treatments with four plasmids, separately. **D** The proliferation ability of ESCC cells were detected by CCK8 assays after treatments with four plasmids, separately. **E** The proliferation ability of ESCC cells were detected by clone formation assay after treatments with four plasmids, separately. **F** The proliferation ability of ESCC cells were detected by Edu assays after treatments with four plasmids, separately (bar = 100 μm). **G** Cell cycle changes in ESCC cells were assessed by flow cytometry after treatments with four plasmids, separately. **P < 0.01, ***P < 0.001.

To further explore the biological function of circUBE4B-173aa, we compared circUBE4B with the ATG mutation (circUBE4B-ATG-mut-Flag), which would inhibit the translation of circUBE4B-173aa, and the linear form of circUBE4B (line-circUBE4B-173aa-Flag), which would encode circUBE4B-173aa (Fig. 4A). The qRT-PCR results showed that circUBE4B was upregulated significantly expressed in the ESCC cells transfected with circUBE4B-Flag plasmids, circUBE4B-ATG-mut-Flag plasmids, and line-circUBE4B-173aa-Flag plasmids, while linear form of UBE4B mRNA expression was not obviously changed in the above groups of ESCC cells (Fig. 4B). Additionally, Western Blot showed that circUBE4B-173aa were detected in circUBE4B-Flag group and line-circUBE4B-Flag group, but not in circUBE4B-ATG-mut-Flag group. Furthermore, we found that the expression levels of the parental protein UBE4B were not changed in these groups (Fig. 4C).

Subsequently, we performed CCK8 assays (Fig. 4D) and clone formation assays (Fig. 4E) to evaluate the changes in the proliferation of ESCC cells. The results showed that the proliferation of ESCC cells was significantly enhanced in the circUBE4B-Flag group and line-circUBE4B-173aa-Flag group compared with a control group and circUBE4B-ATG-mut-Flag group. These results showed that only overexpression of circUBE4B-173aa protein could enhance the proliferation of ESCC cells. Similar results were confirmed by the Edu experiment (Fig. 4F). Flow cytometry results showed that the proportion of ESCC cells in the S-phase increased significantly in the circUBE4B-Flag group and line-circUBE4B-173aa-Flag group, while the proportion of ESCC cells in S-phase did not change significantly in the control group and circUBE4B-ATG-mut-Flag group (Fig. 4G). Taken together, these results suggested that the promotion of the proliferative capacity in the ESCC cells caused by circUBE4B depends on its encoding circUBE4B-173aa protein.

### CircUBE4B-173aa interacts with MAPK1 to activate the MAPK/ERK pathway

**Fig. 5:**
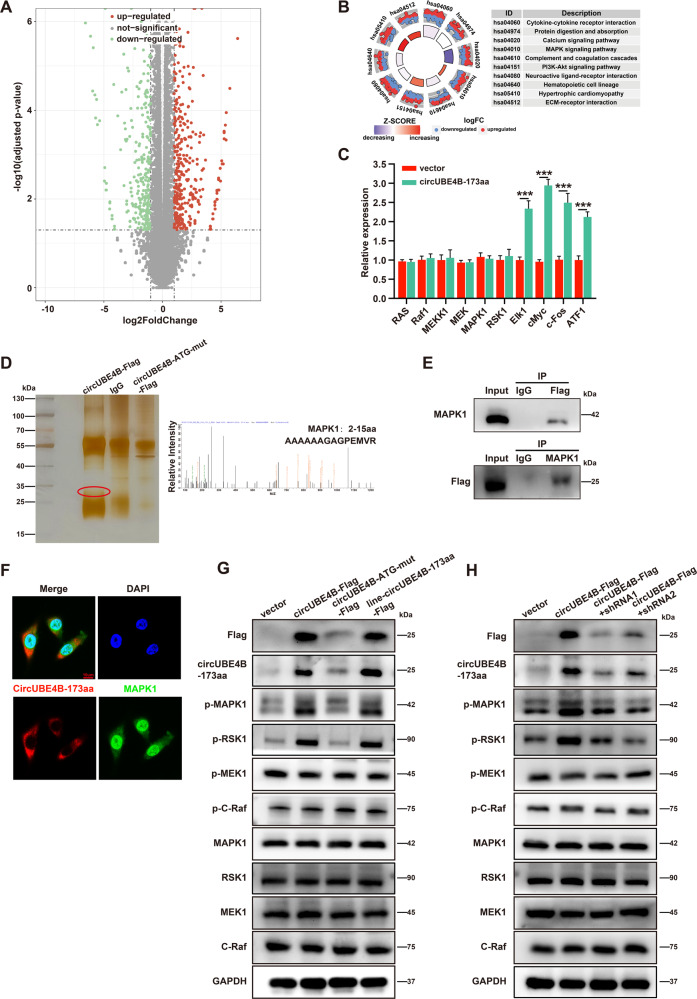
CircUBE4B-173aa interacts with MAPK1 to activate the MAPK/ERK pathway. **A** The volcano plot shows the RNA-seq sequencing results after overexpression of linear-circUBE4B-173aa-Flag. The red dots indicate genes with significantly upregulated expression, the green dots indicate genes with significantly downregulated expression, and the gray dots indicate genes with insignificant expression differences. **B** The bubble diagram shows the up- and down-regulation of the signaling pathways that underwent significant changes in top ten of ESCC cells overexpressed linear-circUBE4B-173aa-Flag. **C** qRT-PCR analysis of the relative expression changes of key genes in the MAPK pathway in ESCC cells overexpressed linear-circUBE4B-173aa-Flag. **D** Left, immunoprecipitation assays were performed using anti-immunoglobulin G (IgG) or anti-Flag antibodies in ESCC cells overexpressed linear-circUBE4B-173aa-Flag or circUBE4B-173aa-ATG-mut-Flag. The precipitated proteins were separated by Western Blot assay and then visualized in combination with silver staining assay. Protein glue strips with differential expression were sent for liquid chromatography-tandem mass spectrometry (LC-MS/MS) analysis to identify potential proteins interacting with circUBE4B-173aa. Right, MAPK1 was identified by LC-MS/MS in the circUBE4B-173aa protein complexes. **E** Co-IP experiments show the mutual interaction of MAPK1 and circUBE4B-173aa. **F** Immunofluorescence staining showed co-localization of circUBE4B-173aa with MAPK1 protein at the cytoplasm in ESCC cells (bar = 10 μm). **G** The expression of total and phosphorylated MAPK1, RSK1, and MEK1 were examined by Western Blot in ESCC cells after treatments with four plasmids, separately. **H** The expression of total and phosphorylated MAPK1, RSK1, and MEK1 were examined by Western blot in ESCC cells after co-transfection with circUBE4B-Flag plasmids and circUBE4B shRNA plasmids. ***P < 0.001.

To further elucidate the potential mechanism by which circUBE4B-173aa promotes the proliferation of ESCC cells, RNA-seq was used to detect gene expression profiles in ESCC cells overexpression of circUBE4B-173aa compared to the control group. The variation of gene expression was demonstrated in the volcano plot (Fig. 5A). KEGG analysis (Fig. S2A), and simultaneously Gene Set Enrichment Analysis (GSEA) (Fig. 5B) revealed that the differential expressed genes were enriched in the MAPK pathway. The MAPK pathway plays a critical role in the survival, differentiation, and drug resistance of tumor cells [20]. Consistently, the mRNA levels of downstream genes of the MAPK pathway were increased after overexpression of circUBE4B-173aa (Fig. 5C). To determine the downstream target of circUBE4B-173aa that contributes to the MAPK pathway, IP of circUBE4B-173aa followed by mass spectrometry analyses was carried out to identify the proteins binding to circUBE4B-173aa. Surprisingly, among the detected proteins, the MAPK1 protein was identified (Fig. 5D and Fig. S2B). ERK1 (MAPK3) and ERK2 (MAPK1) have been reported to be one of the most important protein kinases involved in Ras-Raf-MEK-ERK signaling [21]. To verify the interaction between MAPK1 and circUBE4B-173aa, Western Blot was carried out to analyze the immunocomplex of circUBE4B-173aa. The results clearly demonstrated that MAPK1 was indeed interacted with by circUBE4B-173aa in ESCC cells (Fig. 5E). The MAPK1 protein was separated into three fragments with hemagglutinin (HA) tags to further explore the region of MAPK1 interacted with circUBE4B-173aa (Fig. S2D) and the immunoprecipitation assays were confirmed that circUBE4B-173aa mainly interacted with the middle region (121aa–240aa) of MAPK1 (Fig. S2E). Next, we explored the distribution of circUBE4B-173aa and MAPK1 in ESCC cells. Nuclear and cytoplasmic protein extraction showed that circUBE4B-173aa was mainly located in the cytoplasm, while the MAPK1 protein was located in the nucleus and cytoplasm (Fig. S2C). Furthermore, the co-localizations between circUBE4B-173aa and MAPK1 were confirmed in the cytoplasmic of ESCC cells by immunofluorescence assays (Fig. 5F).

It’s known that the translocation and transcriptional activity of MAPK1 depends on its phosphorylation. We next explored whether the activity of MAPK1 was regulated by circUBE4B-173aa. Western Blot showed that the phosphorylation levels of MAPK1 and RSK1 were remarkably upregulated in circUBE4B-Flag and line-circUBE4B-173aa-Flag overexpressing ESCC cells. Nevertheless, no significant changes were observed in total MAPK1 protein as well as expression levels of total C-Raf, MEK1, and RSK1 protein in the control group and circUBE4B- ATG-mut-Flag group (Fig. 5G) and the depletion of circUBE4B inhibited the phosphorylation of MAPK1 and RSK1 (Fig. 5H). Of note, neither depletion nor overexpression of circUBE4B affected the total level of MAPK1 or RSK1. Overall, the results suggest that circUBE4B-173aa may play a cancer-promoting role by binding to MAPK1 and then activating the MAPK/ERK signaling pathway.

### CircUBE4B-173aa promote tumorigenesis in vivo

**Fig. 6:**
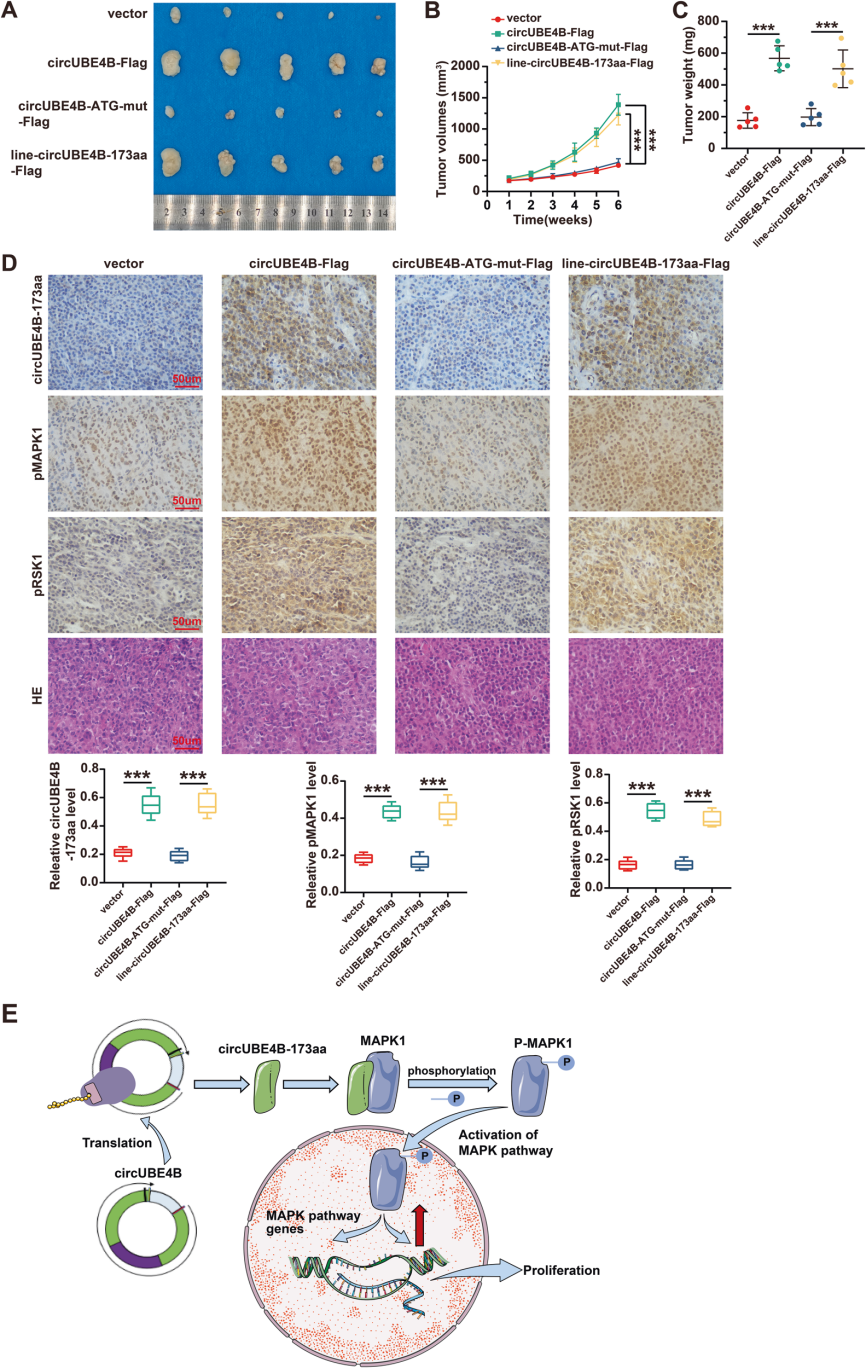
CircUBE4B-173aa promote tumorigenesis in vivo. **A** Images of Xenograft tumors in each group of BALB/c Nude mice at the end of the experiment (*n* = 5 per group). **B** Volume growth curves of xenograft tumors in mice. Tumor volumes were measured every 7 days after inoculation (*n* = 5 per group, unit: mm^3^) for a total period of 6 weeks. **C** The weight of xenograft tumors in mice were measured at the end of the experiment (*n* = 5 per group, unit: mg). **D** Top: IHC and HE staining results of circUBE4B-173aa, phosphorylated MAPK1, and phosphorylated RSK1 in mouse xenograft tumors (*n* = 5 mice per group). Bottom: Diagram showing the IHC results for circUBE4B-173aa, phosphorylated MAPK1, and phosphorylated RSK1 expression in mouse xenograft tumors (bar = 50 μm). **E**. Schematic diagram describing the molecular mechanism by which circUBE4B-173aa encoded by circUBE4B promotes progression of ESCC by augmenting MAPK/ERK signaling. ***P < 0.001.

To further investigate whether circUBE4B contributed to tumor progression in vivo, ESCC cells with circUBE4B overexpression were grafted to the flank of nude mice. As shown in Fig. 6A, tumors derived from circUBE4B-Flag overexpression and line-circUBE4B-173aa-Flag were larger. Both the tumor growth rate (Fig. 6B) and tumor weight (Fig. 6C) of circUBE4B-Flag and line-circUBE4B-173aa-Flag overexpression groups were significantly higher than control and circUBE4B-ATG-mut-Flag group. Next, the abundance of circUBE4B and phosphorylation levels of MAPK1 and RSK1 were assessed by immunohistochemical (IHC) staining. Statistical analyses have consistently demonstrated that the expression of a circUBE4B-173aa protein, phosphorylation of MAPK1 and RSK1 were significantly higher in circUBE4B-Flag and line-circUBE4B-173aa-Flag overexpression tumors than that in control and circUBE4B-ATG-mut-Flag tumors (Fig. 6D). Our results confirm that circUBE4B-173aa is also cancer-promoting in xenograft animals, which is consistent with our previous experimental results in vitro.

## Discussion

More and more studies have shown that circRNAs can be widely involved in multiple biological processes and diseases by acting as miRNA sponges or interacting with protein complexes [22]. We reported previously that circGSK3β promoted ESCC cell migration and invasion via directly interacting with GSK3β and inhibiting GSK3β activity [16], and hsa_circ_0006948 enhances ESCC progression and epithelial-mesenchymal transition through the miR-490-3p sponge [17], indicating that circRNA plays an important role in the development of ESCC and regulates cancer-related pathways. With the deepening of research, a small subset of circRNAs are translatable using internal ribosome entry site (IRES)-mediated [23] or N^6^-meth-yladenosine (m^6^A)-dependent [24] translation initiation. Recent studies have shown that some circRNAs and their circRNA-encoded proteins(circ-proteins) are involved in regulating tumor progression, chemotherapy resistance, invasion and migration, which shows great latent capacity of application in early tumor diagnosis and clinical prognosis [22, 25–29]. Meanwhile, the unique loop-like structure of circRNA makes itself more stable in cells [30], and it might be able to consistently translate functional proteins in a stable state. However, the role of circRNA encoding proteins in esophageal cancer have not been reported.

In this study, we identified a novel circRNA, which is upregulated in ESCC. Overexpression of circUBE4B was significantly associated with larger tumor size, lower tumor differentiation, and poor prognosis in ESCC patients. Furthermore, in vitro and in vivo experiments showed that the proliferation of ESCC cells was significantly inhibited after the depletion of circUBE4B. Further studies confirmed that circUBE4B encoded a novel protein, circUBE4B-173aa. As we know, circRNA functions as a miRNA sponge and interacts with proteins directly, or translation of circRNA-specific peptides. In our previous experiment, RNA-binding protein Immunoprecipitation (RIP) revealed that circUBE4B was not associated with the Argonaute-2 (AGO2) protein, a key component of the microRNA-containing RISC complex. Therefore, circUBE4B might not exert its function through the binding and interception of miRNAs. Then we mutated the start codon of circUBE4B and constructed the linear form of the ORF sequence in circUBE4B in ESCC cells. We found that the tumor-promotion effects were almost lost, indicating that these effects mainly functioned by translating circUBE4B-173aa. More importantly, an exclusive antibody targeting circUBE4B-173aa has been designed and applied to assess the expressions of circUBE4B-173aa in ESCC patients. The results showed that the overexpression level of circUBE4B-173aa was significantly correlated with larger tumor size, lower tumor differentiation, and poor outcome in ESCC patients. Therefore, the results, for the first time, demonstrate that circUBE4B is a potent tumor promoter and that the abundance of circUBE4B has the potential to serve as a biomarker for ESCC prognosis.

The MAPK cascade is an important and conserved evolutionary signaling pathway involved in regulating normal cell proliferation, survival and differentiation, and other life processes [31]. It is known that aberrant activation of the MAPK pathway is also present in ESCC [32]. To deeply analyze the downstream signaling pathways regulated by circUBE4B-173aa, we performed RNA-seq analysis in ESCC cells overexpressed circUBE4B-173aa and pathway enrichment analysis for gene expression profiles. Surprisingly, we found that the MAPK pathway was significantly activated in ESCC cells after overexpression of circUBE4B-173aa. Then, we performed co-immunoprecipitation(co-IP) assays and mass spectrometry and found that MAPK1 (ERK2) protein interacted with circUBE4B-173aa. Further experiments suggest that the phosphorylation level of MAPK1 protein and RSK1 were upregulated in ESCC cells overexpressed circUBE4B-173aa, indicating that the MAPK/ERK pathway was activated. Taken together, the circUBE4B-173aa protein interacts with MAPK1 and promotes the phosphorylation level of MAPK1 to eventually activate MAPK/ERK signaling pathway.

With the development of research, numerous individual circRNAs have been shown to be differentially expressed in tumors relative to adjacent non-malignant tissues and associated with certain clinical characteristics, such as tumor size, histological grade, and tumor, node, metastasis (TNM) stage [22, 33]. Such findings highlight circRNAs as promising diagnostic and prognostic biomarkers, which is further substantiated by their high stability and detectability in biofluids (such as plasma, saliva, and urine) [34–36]. Fan et al. identified that Hsa_circ_0001946 and hsa_circ_0043603 as potential diagnostic biomarkers in plasma and secreted by exosomes in ESCC [37]. In addition, emerging evidence indicated the potential diagnostic, predictive, and therapeutic value of tumor-related functional peptides encoded by circRNAs [38]. The field of circRNA research has witnessed an immense number of new studies, which have elucidated diverse mechanisms of action and functional roles in tumorigenesis. But most of them still focus on the applied basic research, and there are no circRNA-related products successfully applied in the clinical cases, especially in the esophageal carcinoma yet. However, we devote to the clinical transformation of circRNA, and surprisingly, it is the first time to detect the existence of circRNA-translated protein in the ESCC tumor tissue by our original antibody. More precisely, it is possible for the expression level of circUBE4B-173aa in tumor tissues to serve as a biomarker for ESCC prognosis.

In summary, our study demonstrated that circUBE4B encoding circUBE4B-173aa ultimately promotes ESCC proliferation by interacting with MAPK1 protein and activating the MAPK/ERK pathway (Fig. 6E), which indicated that circUBE4B/circUBE4B-173aa can be used as a potential prognosis biomarker and new therapeutic targets for ESCC. However, it is prompt for us to elucidate the detailed molecular mechanism that circUBE4B-173aa interacts with MAPK1 and promotes its phosphorylation level in further study. Meanwhile, more studies are urgently needed to confirm the valuable clinical utility of circUBE4B/circUBE4B-173aa for ESCC in more depth.

## Materials and methods

### Human samples

Patients who underwent surgery and eventually diagnosed by histopathology with esophageal squamous cell carcinoma in 2013–2015 were recruited for this study. ESCC tissue and paired paraneoplastic tissues were collected from each patient for further experiments such as qPCR and Western Blot analysis. All patients did not receive preoperative radiotherapy or chemotherapy. This study was approved by the Medical Ethics Committee of Sun Yat-sen Memorial Hospital, Sun Yat-sen University. Written informed consent was obtained from each patient prior to this study. The inclusion criteria for this study were as follows: (1) Patients were confirmed to have ESCC by postoperative histopathology. (2) Patients did not receive radiofrequency ablation, chemotherapy or targeted drugs before surgery. (3) Patients without a history of other tumors. The exclusion criteria for this study were as follows: (1) Patients were diagnosed with esophageal adenocarcinoma and esophageal benign lesions. (2) Patients with incomplete clinical information or follow-up data. All patients who participated in this study were followed up continuously. The clinicopathological characteristics of the patients are listed in Tables 1, 2. The raw data of circUBE4B/circUBE4B-173aa expression for each patient have been attached to Supplementary Material 2.

### Microarray analysis

Three pairs of ESCC tissues and adjacent normal tissues were analyzed for differentially expressed circRNA using a circRNA expression microarray (Aksomics). All raw data from the microarray analysis have been uploaded to Gene Expression Omnibus, accession number GSE131969.

### Cell culture and RNase R treatment

HEK293T cells, human ESCC cells (TE1, Eca109, KYSE-150, and KYSE-180), and normal esophageal epithelial cells (HEEC) were purchased from the Cell Bank of the Chinese Academy of Sciences (Shanghai, China). TE1, Eca109, KYSE-150, and KYSE-180 were cultured with RPMI-1640 medium (Gibco, MA, USA) containing 10% FBS (Gibco, MA, USA) and 1% penicillin/streptomycin (Millipore). HEK293T cells were cultured in a DMEM medium (Gibco, MA, USA) containing 10% FBS and 1% penicillin/streptomycin (Millipore). The cell lines were authenticated by Short Tandem Repeat profiling. RNase R (Epicentre Technologies, USA) was used to degrade linear mRNA. Briefly, we extracted RNA from TE1 and Eca109 cells and divided the RNA into two groups, one group digested by RNase R and the other group as a control was treated with digestion buffer only. The samples were incubated at 37 °C for 30 min, and then the expression levels of circUBE4B and UBE4B were detected by real-time quantitative qRT-PCR.

### Total RNA extraction and quantitative real-time PCR

Total RNA samples were extracted using Trizol (TIANGEN) according to the manufacturer’s instructions. The concentrations and purities of all samples were subsequently measured by NanoDrop2000 (Thermo Scientific, Wilmington, DE, USA). The corresponding cDNAs were reverse transcribed using PrimeScript RT Master Mix (TaKaRa, Shiga, Japan). qPCR SYBR Green Master Mix (Yeasen, Shanghai, China) was used to prepare the qRT-PCR reaction system, and the PCR reactions were performed on a LightCycler®96 system (Roche, Switzerland). The qRT-PCR reaction was performed at 95 °C for 5 min, followed by 40 cycles of 95 °C for 10 s and a primer-specific annealing temperature of 60 °C for 30 s. GAPDH was used as an internal reference and all samples were replicated three times. Relative expression of the genes was calculated using the ΔCq method [39]. All primer sequences are given in Table S4.

### RNA fluorescence in situ hybridization

RNA fluorescence in situ hybridization (RNA-FISH) experiments were performed by fluorescence in situ hybridization kit (RiboBio, Guangzhou, China) according to the manufacturer’s instructions. 5’-Cy3-labeled probes targeting the reverse splice site of circUBE4B were used to determine the subcellular localization of circUBE4B. Images were acquired using a Zeiss LSM 710 laser scanning confocal system (Zeiss, Oberkochen, Germany).

### Plasmids and cell transfection

The short hairpin RNA (shRNA) targeting circUBE4B was purchased from GeteinBiotech, Guangzhou, China. CircUBE4B overexpression vector and control plasmid (pLV-CiR-CMV-MCS-EF1a-GFP-puro) were purchased from GeteinBiotech, Guangzhou, China. To verify the presence and function of circUBE4B-173aa, we constructed four Flag-tagged circUBE4B lentiviral plasmids (vector, circUBE4B-Flag, circUBE4B-ATG-mut-Flag, and line-circUBE4B-173aa-Flag) and stably transfected them into ESCC cells. To verify the interaction region of circUBE4B-173aa and MAPK1, we constructed four HA-tagged MAPK1 plasmids (MAPK1-full length, MAPK1-truncated-1, MAPK1-truncated-2, and MAPK1-truncated-3), and circUBE4B-Flag plasmids and each HA-tagged MAPK1 plasmids were cotransfected into ESCC cells. The transfection was performed when the cells grew to ~70% density, after which stable expression cells were obtained by puromycin screening. The complete sequence of circUBE4B overexpression vectors, circUBE4B knockdown vectors and HA-tagged MAPK1 vectors are shown in Tables S5– S7.

### Cell proliferation assay

Cell Counting Kit-8 (CCK8, ImmunoWay Biotechnology Company Plano, TX, USA) was used to detect the growth capacity of ESCC cells. Cells transfected with sh-circUBE4B or overexpression plasmids were inoculated into 96-well plates and the OD450 values were measured after 24, 48, 72, 96, and 120 h of incubation, respectively.

### Clone formation assay

A clone formation assay was used to evaluate the cloning ability of ESCC cells. The transfected cells were inoculated in six-well plates and cultured for 2 weeks. The cells were fixed with 4% paraformaldehyde for 30 min, and then stained with crystal violet solution for 20 min. Finally, the visible cell clones were subjected to image acquisition, counting, and statistical analysis.

### Flow cytometry

The cell cycle was detected using a BD FACSCANTO™ II flow cytometer according to the instructions of the Beyotime Cell Cycle Kit (Beyotime, Shanghai, China). The results were analyzed with ModFit LT software (Verity Software House, Los Angeles, USA).

### EdU assay

Cell proliferation status was detected using the BeyoClick EdU Cell Proliferation Kit with Alexa Fluor 555 (Beyotime, Shanghai, China) according to the reagent manufacturer’s instructions. Images were acquired using an Olympus IX73 inverted fluorescence microscope system (Olympus, Tokyo, Japan).

### Dual-luciferase reporter assay

The predicted IRES full-length sequences, mutated sequences, and truncated sequences were inserted into pcDNA3.1(+)-RLuc-MCS-Luc vector (GeteinBiotech, Guangzhou, China) and the reporter vector was transfected into 293T cells. Firefly and sea kidney fluorophore lyase activities were measured using the Dual-Glo fluorophore lyase assay kit (Vazyme, Nanjing, China). The complete sequence of dual-luciferase reporter genes of IRES in circUBE4B are shown in Table S8.

### Co-immunoprecipitation and mass spectrometry

Immunoprecipitation experiments were performed using the Co-Immunoprecipitation (IP/Co-IP) kit (Absin, Shanghai, China). Briefly, transfected cells were harvested, washed three times with PBS, and then crushed in RIPA buffer for 30 min at 4 °C. Precipitation was removed from cell lysates by centrifugation (14,000×*g* for 10 min), and supernatants were pre-cleared with protein A/G beads for 1 h. The pre-clearance protein A/G beads were removed, and the target antibody was added for overnight incubation. New protein A/G beads were then added for immunoprecipitation. The final precipitates were collected and detected by Western Blot analysis with the indicated antibodies. The protein bands were visualized by silver staining, and bands were manually excised from the gel and digested with sequencing-grade trypsin (Promega, Madison, WI, USA). The digested peptides were analyzed using a QExactive mass spectrometer (Thermo Fisher Scientific, Carlsbad, CA, USA). Fragment spectra were analyzed using the National Center for Biotechnology Information nonredundant protein database with Mascot (Matrix Science, Boston, MA, USA). The results of mass spectrometry have been attached to Figs.S1B, S2B, and the list of proteins interacted with circUBE4B-173aa has been attached to Supplementary Material 1.

### RNA sequencing

Total RNAs were extracted from ESCC cells overexpression of circUBE4B-173aa and control ESCC cells using TRIzol reagent (TIANGEN, Beijing, China), digested with RNAase R (Epicentre Biotechnologies, Madison, WI) and then treated using the RiboMinus Eukaryote Kit (Qiagen, Valencia, CA) to remove rRNAs. After the rRNAs removed, RNAs was reversed into cDNA using random primers. The cDNA fragments were purified with a QiaQuick PCR extraction kit and ligated to Illumina sequencing adapters. Then the second-strand cDNA was digested with UNG (uracil-*N*-glycosylase), size-selected via agarose gel electrophoresis, and PCR-amplified. Next, the RNA-seq library was deep sequenced with the Illumina HiSeq 2000. RNA sequencing reads were aligned to the human reference genome by the software STAR and RNA abundance was quantified using the software RSEM. RNA sequencing and analysis were performed by Guangzhou Huayin Health Medical Group Cooperation, Guangzhou, China. All the software used to analyze the RNA-seq data are open-source codes from GitHub and the list of the link has been attached to the Table S9.

### Western blot analysis

RIPA buffer (CWBIO, China) was used for protein extraction. Samples were electrophoresed by SDS-PAGE and transferred to nitrocellulose membranes. Protein strips were incubated with primary antibody overnight at 4 °C and then with secondary antibody at room temperature for 1 h. Signal detection and image acquisition were performed using a G: BOX gel imaging system (Syngene, USA) after the addition of ECL luminescent solution (Millipore, Germany). The antibodies used in this study are shown in Table S10.

### Xenograft tumorigenesis

PBS suspensions containing 1 × 10^7^ stably transfected Eca109 cells were randomly inoculated on male BALB/c nude mice aged 4–6 weeks. The sample size for each group was selected based on the premise that significant differences could be detected between groups. The sample size was selected based on feasibility and cost. The width and length of the tumors were measured every week and the tumor volume was calculated according to the formula [40]: volume (mm^3^) = (length × width^2^)/2 in a blinded manner. Mice were executed after 6 weeks and the tumors were removed for weighing and photography. The tumor tissues were fixed and used for immunohistochemical staining. All experimental animals were housed in a pathogen-free environment. Animal experiments were approved by the Guangdong Medical Laboratory Animal Center.

### Immunohistochemistry (IHC)

Xenograft tumor tissues were fixed, dehydrated, embedded, and sectioned according to standard procedures. Antigens were retrieved by boiling in the 10 mM citrate buffer for 3 min and then incubated in 3% hydrogen peroxide for 20 min to block endogenous peroxidase. All sections were incubated with primary antibodies at 4 °C overnight. The IHC kit (ZSGB-BIO, Beijing, China) was then used to detect the specifically bound primary antibodies.

### Sanger sequencing

Total RNAs from TE1 cells were extracted by TRIzol (TIANGEN, Beijing, China) and subjected to cDNA synthesis using a PrimeScript RT Master Mix Kit (TaKaRa, Shiga, Japan). The cDNA was amplified by qRT-PCR using a primer specifically targeting circUBE4B. To confirm the head-to-tail splicing, Sanger sequencing was conducted by using sequencing primer 5′-GCTCCAATCCAGGAACAAGC-3′. The qRT-PCR reaction was performed at 95 °C for 5 min, followed by 40 cycles of 95 °C for 10 s and a primer-specific annealing temperature of 60 °C for 30 s. Individual clones were sequenced using Applied Biosystems BigDye terminator mix version 3.1. By contrast, A-to-I calls made from RNA-seq but not verified by Sanger sequencing as A-to-G are referred to as false positives. The heights of different base peaks from the Sanger sequence were measured by ImageJ software.

### Nuclear and cytoplasmic proteins extraction

Nuclear and cytoplasmic proteins were isolated using the Nuclear and Cytoplasmic Protein Extraction Kit (Absin, Shanghai, China). Briefly, ESCC cells were lysed by Cell Fractionation Buffer for 20 min on ice. Then, the supernatants were collected as the cytoplasmic fractions after centrifugation at 4 °C and 15,000×*g* for 10 min. And the pellets were lysed by Nuclear Isolation Buffer for 5 min on ice. Finally, the supernatants were collected as the nuclear fractions after centrifugation at 4 °C and 15000×*g* for 10 min. The extracted protein samples were subsequently separated on SDS-PAGE gels and analyzed by WB assays. GAPDH was used as the endogenous cytoplasmic control and Histone H3 was used as the endogenous nuclear control.

### Statistical analysis

The data were expressed as mean ± SD of triplicate. Comparison of non-normal data were analyzed using Mann–Whitney *U*-test, and analysis of normalized data were performed using the Student’s *t*-test between two groups. Spearman correlation analysis was applied in analyzing the correlation between the two groups. Overall survival (OS) and progression-free survival (PFS) were estimated by Kaplan–Meier method. All *P* values were based on two-sided testing and statistical analysis was performed using GraphPad Prism 8 (San Diego, USA) statistical software. *P* < 0.05 was considered statistically significant.

## Supplementary information

Supplementary Tables and Figures

Supplementary Material 1

Supplementary Material 2

Original WB

checklist

## Data Availability

All data needed to evaluate the conclusions in this study are presented in the paper. Additional data related to this paper may be requested from the corresponding authors.
