# Supplementary material for: A novel protein encoded by circUBE4B promotes progression of esophageal squamous cell carcinoma by augmenting MAPK/ERK signaling
Source: Cell Death Dis. 2023 Jun 1;14(6):346. doi: 10.1038/s41419-023-05865-2 (PMC10235080; doi:10.1038/s41419-023-05865-2)
Supplement: Supplementary file 1 — Supplementary Tables and Figures [file 41419_2023_5865_MOESM1_ESM.docx]

**A novel protein encoded by circUBE4B** **promotes progression of esophageal squamous cell carcinoma by augmenting MAPK/ERK signaling**

Yingcheng Lyu **^1,2,4^**, Binghua Tan **^1,2,4^**, Lin Li **^1,2,4^**, Ruihao Liang **^1,2^**, Kai Lei **^1,2^**, Kefeng Wang **^1,2^**, Duoguang Wu**^1,2^**, Huayue Lin **^1,3,^ ***, Minghui Wang **^1,2,^ ***

**^1^** Guangdong Provincial Key Laboratory of Malignant Tumor Epigenetics and Gene Regulation, Sun Yat-sen Memorial Hospital, Sun Yat-sen University, 510120 Guangzhou, China.

**^2^** Department of Thoracic Surgery, Sun Yat-sen Memorial Hospital, Sun Yat-sen University, 510120 Guangzhou, China

**^3^** Breast Tumor Center, Sun Yat-sen Memorial Hospital, Sun Yat-sen University, 510120 Guangzhou, China

**^4^** These authors contributed equally: Yingcheng Lyu, Binghua Tan and Lin Li.

*****Corresponding authors: Huayue Lin and Minghui Wang.

E-mail addresses: wmingh@mail.sysu.edu.cn (M. Wang), linhy29@mail.sysu.edu.cn (H. Lin)

## Table S1. The complete list of differentially expressed genes (DEGs) with internal ribosome entry sites in ESCC tumor tissues compared with adjacent tissues.

| **circBase_ID** | **mRNA**  **gene symbol** | **Log_2_(FC)** | **P value** | **Regulation** | **IRES**  **(Y/N)** |
| --- | --- | --- | --- | --- | --- |
| **hsa_circ_0005199** | **UBE4B** | **3.807712** | **0.0052148** | **UP** | **Y** |
| hsa_circ_0058514 | AGFG1 | 3.749795 | 0.0019545 | UP | Y |
| hsa_circ_0038138 | ABCC1 | 3.743654 | 0.0046114 | UP | Y |
| hsa_circ_0055945 | UXS1 | 3.742774 | 0.0118444 | UP | Y |
| hsa_circ_0004791 | PHKB | 3.739123 | 0.002301 | UP | Y |
| hsa_circ_0003410 | UBAP2 | 3.537893 | 0.0138616 | UP | Y |
| hsa_circ_0007693 | ERI3 | 3.474498 | 0.003313 | UP | Y |
| hsa_circ_0002037 | XPO5 | 3.431241 | 0.0097073 | UP | Y |
| hsa_circ_0005771 | PTPRK | 3.400305 | 0.0603719 | UP | Y |
| hsa_circ_0039261 | PHKB | 3.370451 | 0.0006481 | UP | Y |
| hsa_circ_0070022 | SDAD1 | 3.36386 | 0.0168832 | UP | Y |
| hsa_circ_0002688 | WHSC1 | 3.300847 | 0.0278616 | UP | Y |
| hsa_circ_0005535 | SNX13 | 3.283296 | 0.0006983 | UP | Y |
| hsa_circ_0004365 | SEMA3C | 3.267455 | 0.0290216 | UP | Y |
| hsa_circ_0000698 | PHKB | 3.253928 | 0.0077171 | UP | Y |

FC: fold change

Y: YES

N: NO

## Table S2. The correlation analysis between circUBE4B expressions and clinicopathological characteristics.

|  |  | **circUBE4B expression** | **Tumor size** |
| --- | --- | --- | --- |
| **circUBE4B expression** | Spearman Correlation | 1 | 0.348 |
|  | Sig.(2-tailed) |  | **0.02** |
|  | N | 80 | 80 |
| **Tumor size** | Spearman Correlation | 0.348 | 1 |
|  | Sig.(2-tailed) | **0.02** |  |
|  | N | 80 | 80 |
|  | Correlation is significant at the 0.01 level (2-tailed) | |  |
|  |  |  |  |
|  |  | **circUBE4B expression** | **Differentiation** |
| **circUBE4B expression** | Spearman Correlation | 1 | 0.308 |
|  | Sig.(2-tailed) |  | **0.043** |
|  | N | 80 | 80 |
| **Differentiation** | Correlation | 0.308 | 1 |
|  | Sig.(2-tailed) | **0.043** |  |
|  | N | 80 | 80 |
|  | Correlation is significant at the 0.01 level (2-tailed) | |  |

## Table S3. The correlation analysis between circUBE4B-173aa expressions and clinicopathological characteristics.

|  |  | **circUBE4B-173aa expression** | **Tumor size** |
| --- | --- | --- | --- |
| **circUBE4B-173aa expression** | Spearman Correlation | 1 | 0.665 |
|  | Sig.(2-tailed) |  | **<0.001** |
|  | N | 40 | 40 |
| **Tumor size** | Pearson Correlation | 0.665 | 1 |
|  | Sig.(2-tailed) | **<0.001** |  |
|  | N | 40 | 40 |
|  | Correlation is significant at the 0.01 level (2-tailed) | |  |

|  |  | | **circUBE4B-173aa expression** | | **Differentiation** |
| --- | --- | --- | --- | --- | --- |
| **circUBE4B-173aa expression** | Spearman Correlation | | 1 | | 0.293 |
|  | Sig.(2-tailed) | |  | | **0.047** |
|  | N | | 40 | | 40 |
| **Differentiation** | Pearson Correlation | | 0.293 | | 1 |
|  | Sig.(2-tailed) | | **0.047** | |  |
|  | N | | 40 | | 40 |
|  | | Correlation is significant at the 0.01 level (2-tailed) | |  | |

## Table S4. Primer sequences for qRT-PCR.

| **Gene name** | **Primer** | **Sequence (5'-3')** |
| --- | --- | --- |
| GAPDH | Forward | GGAGCGAGATCCCTCCAAAAT |
|  | Reverse | GGCTGTTGTTCATACTTCTCATGG |
| circUBE4B (Divergent primers) | Forward | GCTCCAATCCAGGAACAAGC |
| circUBE4B (Divergent primers) | Reverse | CGATGGGCTACTCCTAGACA |
| circUBE4B  (Convergent primers) | Forward | GATGAGACGCGTGTGAATGC |
| circUBE4B  (Convergent primers) | Reverse | CGGATATAGCGACGGCAACT |
| UBE4B  (Divergent primers) | Forward | GCTCCAATCCAGGAACAAGC |
| UBE4B  (Divergent primers) | Reverse | CGATGGGCTACTCCTAGACA |
| UBE4B  (Convergent primers) | Forward | GATGAGACGCGTGTGAATGC |
| UBE4B  (Convergent primers) | Reverse | CGGATATAGCGACGGCAACT |
| RAS | Forward | GGACTGGGGAGGGCTTTCT |
|  | Reverse | GCCTGTTTTGTGTCTACTGTTCT |
| Raf1 | Forward | CCGAACAAGCAAAGAACAGTG |
|  | Reverse | GACGCAGCATCAGTATTCCAAT |
| MEKK1 | Forward | ATAGGGCCTAACTCTTTCCTGAT |
|  | Reverse | ATAGGGCCTAACTCTTTCCTGAT |
| MEK1 | Forward | CAATGGCGGTGTGGTGTTC |
|  | Reverse | GATTGCGGGTTTGATCTCCAG |
| MAPK1 | Forward | TCACACAGGGTTCCTGACAGA |
|  | Reverse | ATGCAGCCTACAGACCAAATATC |
| Elk1 | Forward | TGACGGAACACATAGCAACTTC |
|  | Reverse | AAGCCATCGGAACAGTAGACT |
| cMYC | Forward | GGCTCCTGGCAAAAGGTCA |
|  | Reverse | CTGCGTAGTTGTGCTGATGT |
| c-Fos | Forward | GGGGCAAGGTGGAACAGTTAT |
|  | Reverse | CCGCTTGGAGTGTATCAGTCA |
| ATF1 | Forward | AGGACTCATCCGACAGCATAG |
|  | Reverse | TTCTGCCCCGTGTATCTTCAG |
| CircUBE4B-FISH-probe |  | Cy3-TTGCATGGGCTACTCCTAGA  CAAAGAGCTGGCA |

## Table S5. The complete sequence of circUBE4B overexpression vectors.

**(1)** **circUBE4B-Flag**

GAGTAGCCCATCGAAGCCAGAGCAGgactacaaagaccatgacggtgattataaagatcatgacatcgattacaaggatgacgatgacaag**TGA**AGGAGTCAGTTCTCTCAGCAGCTCGCCCTCTAATAGCCTTGAAACGCAATCTCAGTCTCTCTCACGTTCCCAGAGCATGGATATCGATGGTGTCTCATGTGAGAAAAGCATGTCCCAGGTGGATGTGGATTCAGGAATTGAAAAC**ATG**GAGGTTGATGAAAATGATCGAAGAGAAAAGCGGAGCCTCAGTGATAAGGAGCCTTCCTCGGGCCCTGAAGTGTCTGAAGAGCAGGCCTTACAGCTGGTCTGTAAGATCTTCCGTGTCTCTTGGAAGGACCGGGACAGAGATGTCATCTTTCTTTCTTCTCTTTCTGCACAGTTTAAGCAGAACCCAAAAGAAGTATTCTCCGATTTTAAGGACTTGATTGGCCAGATTTTAATGGAAGTGCTAATGATGTCCACTCAGACCAGAGATGAAAACCCATTTGCCAGTCTGACAGCCACATCACAGCCAATTGCTGCAGCAGCACGGTCACCAGACAGAAATCTCTTGCTAAACACTGGCTCCAATCCAGGAACAAGCCCCATGTTCTGCAGCGTGGCTTCCTTTGGTGCCAGCTCTTTGTCTAG

**(2) circUBE4B-ATG-mut-Flag**

**ATG→ACG**

GAGTAGCCCATCGAAGCCAGAGCAGgactacaaagaccatgacggtgattataaagatcatgacatcgattacaaggatgacgatgacaag**TGA**AGGAGTCAGTTCTCTCAGCAGCTCGCCCTCTAATAGCCTTGAAACGCAATCTCAGTCTCTCTCACGTTCCCAGAGC**ACG**GATATCGATGGTGTCTCATGTGAGAAAAGCATGTCCCAGGTGGATGTGGATTCAGGAATTGAAAACATGGAGGTTGATGAAAATGATCGAAGAGAAAAGCGGAGCCTCAGTGATAAGGAGCCTTCCTCGGGCCCTGAAGTGTCTGAAGAGCAGGCCTTACAGCTGGTCTGTAAGATCTTCCGTGTCTCTTGGAAGGACCGGGACAGAGATGTCATCTTTCTTTCTTCTCTTTCTGCACAGTTTAAGCAGAACCCAAAAGAAGTATTCTCCGATTTTAAGGACTTGATTGGCCAGATTTTAATGGAAGTGCTAATGATGTCCACTCAGACCAGAGATGAAAACCCATTTGCCAGTCTGACAGCCACATCACAGCCAATTGCTGCAGCAGCACGGTCACCAGACAGAAATCTCTTGCTAAACACTGGCTCCAATCCAGGAACAAGCCCCATGTTCTGCAGCGTGGCTTCCTTTGGTGCCAGCTCTTTGTCTAG

**(3) line-circUBE4B-173aa-Flag**

**ATG**GATATCGATGGTGTCTCATGTGAGAAAAGCATGTCCCAGGTGGATGTGGATTCAGGAATTGAAAACATGGAGGTTGATGAAAATGATCGAAGAGAAAAGCGGAGCCTCAGTGATAAGGAGCCTTCCTCGGGCCCTGAAGTGTCTGAAGAGCAGGCCTTACAGCTGGTCTGTAAGATCTTCCGTGTCTCTTGGAAGGACCGGGACAGAGATGTCATCTTTCTTTCTTCTCTTTCTGCACAGTTTAAGCAGAACCCAAAAGAAGTATTCTCCGATTTTAAGGACTTGATTGGCCAGATTTTAATGGAAGTGCTAATGATGTCCACTCAGACCAGAGATGAAAACCCATTTGCCAGTCTGACAGCCACATCACAGCCAATTGCTGCAGCAGCACGGTCACCAGACAGAAATCTCTTGCTAAACACTGGCTCCAATCCAGGAACAAGCCCCATGTTCTGCAGCGTGGCTTCCTTTGGTGCCAGCTCTTTGTCTAGGAGTAGCCCATCGAAGCCAGAGCAGgactacaaagaccatgacggtgattataaagatcatgacatcgattacaaggatgacgatgacaag**TGA**

**ATG：Translation start site**

**TGA：Translation termination**

## Table S6. The complete sequence of circUBE4B knockdown vectors.

| **Name** | **Sequence (5'-3')** |
| --- | --- |
| circUBE4B-shRNA1- Sense | GCTCTTTGTCTAGGAGTAG |
| circUBE4B-shRNA1-antiSense | CTACTCCTAGACAAAGAGC |
| circUBE4B-shRNA2- Sense | GTCTAGGAGTAGCCCATCG |
| circUBE4B-shRNA2-antiSense | GCATGGGCAACTCCTAGAC |

## Table S7. The complete sequence of HA-tagged MAPK1 vectors.

**1# MAPK1-Full length
CMV-AUG-**GCGGCGGCGGCGGCGGCGGGCGCGGGCCCGGAGATGGTCCGCGGGCAGGTGTTCGACGTGGGGCCGCGCTACACCAACCTCTCGTACATCGGCGAGGGCGCCTACGGCATGGTGTGCTCTGCTTATGATAATGTCAACAAAGTTCGAGTAGCTATCAAGAAAATCAGCCCCTTTGAGCACCAGACCTACTGCCAGAGAACCCTGAGGGAGATAAAAATCTTACTGCGCTTCAGACATGAGAACATCATTGGAATCAATGACATTATTCGAGCACCAACCATCGAGCAAATGAAAGATGTATATATAGTACAGGACCTCATGGAAACAGATCTTTACAAGCTCTTGAAGACACAACACCTCAGCAATGACCATATCTGCTATTTTCTCTACCAGATCCTCAGAGGGTTAAAATATATCCATTCAGCTAACGTTCTGCACCGTGACCTCAAGCCTTCCAACCTGCTGCTCAACACCACCTGTGATCTCAAGATCTGTGACTTTGGCCTGGCCCGTGTTGCAGATCCAGACCATGATCACACAGGGTTCCTGACAGAATATGTGGCCACACGTTGGTACAGGGCTCCAGAAATTATGTTGAATTCCAAGGGCTACACCAAGTCCATTGATATTTGGTCTGTAGGCTGCATTCTGGCAGAAATGCTTTCTAACAGGCCCATCTTTCCAGGGAAGCATTATCTTGACCAGCTGAACCACATTTTGGGTATTCTTGGATCCCCATCACAAGAAGACCTGAATTGTATAATAAATTTAAAAGCTAGGAACTATTTGCTTTCTCTTCCACACAAAAATAAGGTGCCATGGAACAGGCTGTTCCCAAATGCTGACTCCAAAGCTCTGGACTTATTGGACAAAATGTTGACATTCAACCCACACAAGAGGATTGAAGTAGAACAGGCTCTGGCCCACCCATATCTGGAGCAGTATTACGACCCGAGTGACGAGCCCATCGCCGAAGCACCATTCAAGTTCGACATGGAATTGGATGACTTGCCTAAGGAAAAGCTCAAAGAACTAATTTTTGAAGAGACTGCTAGATTCCAGCCAGGATACAGATCTtacccatacgacgtcccagactacgct**-UAA-AAAA**

**2# MAPK1-truncated-1**

**CMV-AUG-**GCGGCGGCGGCGGCGGCGGGCGCGGGCCCGGAGATGGTCCGCGGGCAGGTGTTCGACGTGGGGCCGCGCTACACCAACCTCTCGTACATCGGCGAGGGCGCCTACGGCATGGTGTGCTCTGCTTATGATAATGTCAACAAAGTTCGAGTAGCTATCAAGAAAATCAGCCCCTTTGAGCACCAGACCTACTGCCAGAGAACCCTGAGGGAGATAAAAATCTTACTGCGCTTCAGACATGAGAACATCATTGGAATCAATGACATTATTCGAGCACCAACCATCGAGCAAATGAAAGATGTATATATAGTACAGGACCTCATGGAAACAGATCTTTACAAGCTCTTGAAGACACAACACtacccatacgacgtcccagactacgct**-UAA-AAAA**

**3# MAPK1-truncated-2**

**CMV-AUG-**

CTCAGCAATGACCATATCTGCTATTTTCTCTACCAGATCCTCAGAGGGTTAAAATATATCCATTCAGCTAACGTTCTGCACCGTGACCTCAAGCCTTCCAACCTGCTGCTCAACACCACCTGTGATCTCAAGATCTGTGACTTTGGCCTGGCCCGTGTTGCAGATCCAGACCATGATCACACAGGGTTCCTGACAGAATATGTGGCCACACGTTGGTACAGGGCTCCAGAAATTATGTTGAATTCCAAGGGCTACACCAAGTCCATTGATATTTGGTCTGTAGGCTGCATTCTGGCAGAAATGCTTTCTAACAGGCCCATCTTTCCAGGGAAGCATTATCTTGACCAGCTGAACCACATTtacccatacgacgtcccagactacgct**-UAA-AAAA**

**4# MAPK1-truncated-3**

**CMV-AUG-**

TTGGGTATTCTTGGATCCCCATCACAAGAAGACCTGAATTGTATAATAAATTTAAAAGCTAGGAACTATTTGCTTTCTCTTCCACACAAAAATAAGGTGCCATGGAACAGGCTGTTCCCAAATGCTGACTCCAAAGCTCTGGACTTATTGGACAAAATGTTGACATTCAACCCACACAAGAGGATTGAAGTAGAACAGGCTCTGGCCCACCCATATCTGGAGCAGTATTACGACCCGAGTGACGAGCCCATCGCCGAAGCACCATTCAAGTTCGACATGGAATTGGATGACTTGCCTAAGGAAAAGCTCAAAGAACTAATTTTTGAAGAGACTGCTAGATTCCAGCCAGGATACAGATCTtacccatacgacgtcccagactacgct-**UAA-AAAA**

## Table S8. The complete sequence of Dual-Luciferase Reporter genes of IRES in circUBE4B.

| **Name** | **Sequence (5'-3')** |
| --- | --- |
| **1# Empty vector** | CMV-AUG-**Rluc**-UAA-Empty-AUG-**Luc**-UAA-AAAA |
| **2# IRES1-WT** | CMV-AUG-**Rluc**-UAA-TGTCTGAAGAGCAGGCCTTACAGCTGGTCTGTAAGATCTTCCGTGTCTCTTGGAAGGACCGGGACAGAGATGTCATCTTTCTTTCTTCTCTTTCTGCACAGTTTAAGCAGAACCCAAAAGAAGTATTCTCCGATTTTAAGGACT-AUG-**Luc**-UAA-AAAACTGACAGCCACATCACAGCCAATTGCTGCAGCAGCACGGTCACCAGACAGAAATCTCTTGCTAAACACTGGCTCCAATCCAGGAACAAGCCCCATGTTCTGCAGCGTGGCTTCCTTTGGTGCCAGCTCTTTGTCTAG |
| **3# IRES1-mut** | CMV-AUG-**Rluc**-UAA-  TGTGTGAAGACCAGGCCATACTGCAGGTGTCTAAGATGTTGCCTCTCTCTAGCAAGGACGGGCACAGTGTTCTCATCTTTCTTTCTTCTCTTTCTGCACAGTTTAAGCAGAACCCAAAAGAAGTATTCTCCGATTTTAAGGACT-AUG-**Luc**-UAA-AAAA |
| **4# IRES1-DEL-1** | CMV-AUG-**Rluc**-UAA-TGTCTGAAGAGCAGGCCTTACAGCTGGTCTGTAAGATCTTCCGTGTCTCTTGGAAGGACCGGGACAGAGATG-AUG-**Luc**-UAA-AAAA |
| **5# IRES1-DEL-2** | CMV-AUG-**Rluc**-UAA- TCATCTTTCTTTCTTCTCTTTCTGCACAGTTTAAGCAGAACCCAAAAGAAGTATTCTCCGATTTTAAGGACT-AUG-**Luc**-UAA-AAAA |

## Table S9. The link to the GitHub repository with RNA-seq data analysis.

| **Software** | **Website address** |
| --- | --- |
| **SOAPnuke** | [GitHub - BGI-flexlab/SOAPnuke: A Tool for integrated Quality Control and Preprocessing on FASTQ or BAM/CRAM files](https://github.com/BGI-flexlab/SOAPnuke) |
| **Bowtie** | [GitHub - jwkvam/bowtie: Create a dashboard with python!](https://github.com/jwkvam/bowtie) |
| **Cufflinks** | [GitHub - santosjorge/cufflinks: Productivity Tools for Plotly + Pandas](https://github.com/santosjorge/cufflinks) |
| **RSEM** | [GitHub - deweylab/RSEM: RSEM: accurate quantification of gene and isoform expression from RNA-Seq data](https://github.com/deweylab/RSEM) |
| **GATK** | [GitHub - broadinstitute/gatk: Official code repository for GATK versions 4 and up](https://github.com/broadinstitute/gatk) |
| **EdgeR** | [unistbig/compareDEtools (github.com)](https://github.com/unistbig/compareDEtools) |
| **Clusterprofiler** | [YuLab-SMU/clusterProfiler.dplyr: dplyr verbs for clusterProfiler outputs (github.com)](https://github.com/YuLab-SMU/clusterProfiler.dplyr) |
| **Tophat** | [DaehwanKimLab/tophat: Spliced read mapper for RNA-Seq (github.com)](https://github.com/DaehwanKimLab/tophat) |
| **Soapfuse** | [Nobel-Justin/SOAPfuse: Perl modules of SOAPfuse, a tool for identifying fusion transcripts from paired-end RNA-Seq data. (github.com)](https://github.com/Nobel-Justin/SOAPfuse) |

## Table S10. Antibodies used in this study.

| **Antibody** | **Manufacturers** | **Applications** |
| --- | --- | --- |
| UBE4B | #A301-123A, Thermo Fisher, Waltham, MA, USA | 1:1000 for WB |
| Flag | #NBP2-37823, Novus, SLLouis, MO, USA | 1:1000 for WB  1:500 for IP  1:100 for IF |
| MAPK1/2 | #4695, Cell Signaling Technology, Beverly, MA, USA | 1:1000 for WB  1:50 for IP  1:800 for IF |
| p-MAPK1/2 | #4370, Cell Signaling Technology, Beverly, MA, USA | 1:1000 for WB |
| MEK1/2 | #4694, Cell Signaling Technology, Beverly, MA, USA | 1:1000 for WB |
| p-MEK1/2 | #9154, Cell Signaling Technology, Beverly, MA, USA | 1:1000 for WB |
| RSK1 | #8408, Cell Signaling Technology, Beverly, MA, USA | 1:1000 for WB |
| p-RSK1 | #8753, Cell Signaling Technology, Beverly, MA, USA | 1:1000 for WB |
| c-Raf | #53745, Cell Signaling Technology, Beverly, MA, USA | 1:1000 for WB |
| p-c-Raf | #9421, Cell Signaling Technology, Beverly, MA, USA | 1:1000 for WB |
| GAPDH | #YM3215, Immunoway, CA, USA | 1:1000 for WB |
| Histone H3 | # 4499S, Cell Signaling Technology, Beverly, MA, USA | 1:2000 for WB |
| HA-Tag (C29F4) | #37244，Cell Signaling Technology, Beverly, MA, USA | 1:1000 for WB  1:50 for IP |
| HRP-linked anti-rabbit IgG | #RS0002, Immunoway, CA, USA | 1:10000 for WB |
| HRP-linked anti-mouse IgG | #RS0001, Immunoway, CA, USA | 1:10000 for WB |
| Anti-mouse IgG (H+L) (Alexa Fluor® 594 Conjugate) | #ZF-0513, ZSGB-BIO, Beijing, China | 1:100 for IF |
| Anti-rabbit IgG (H+L) (Alexa Fluor® 488 Conjugate) | #ZF-0513, ZSGB-BIO, Beijing, China | 1:100 for IF |

## Supplemental Figure S1 *related to Figure 3* *
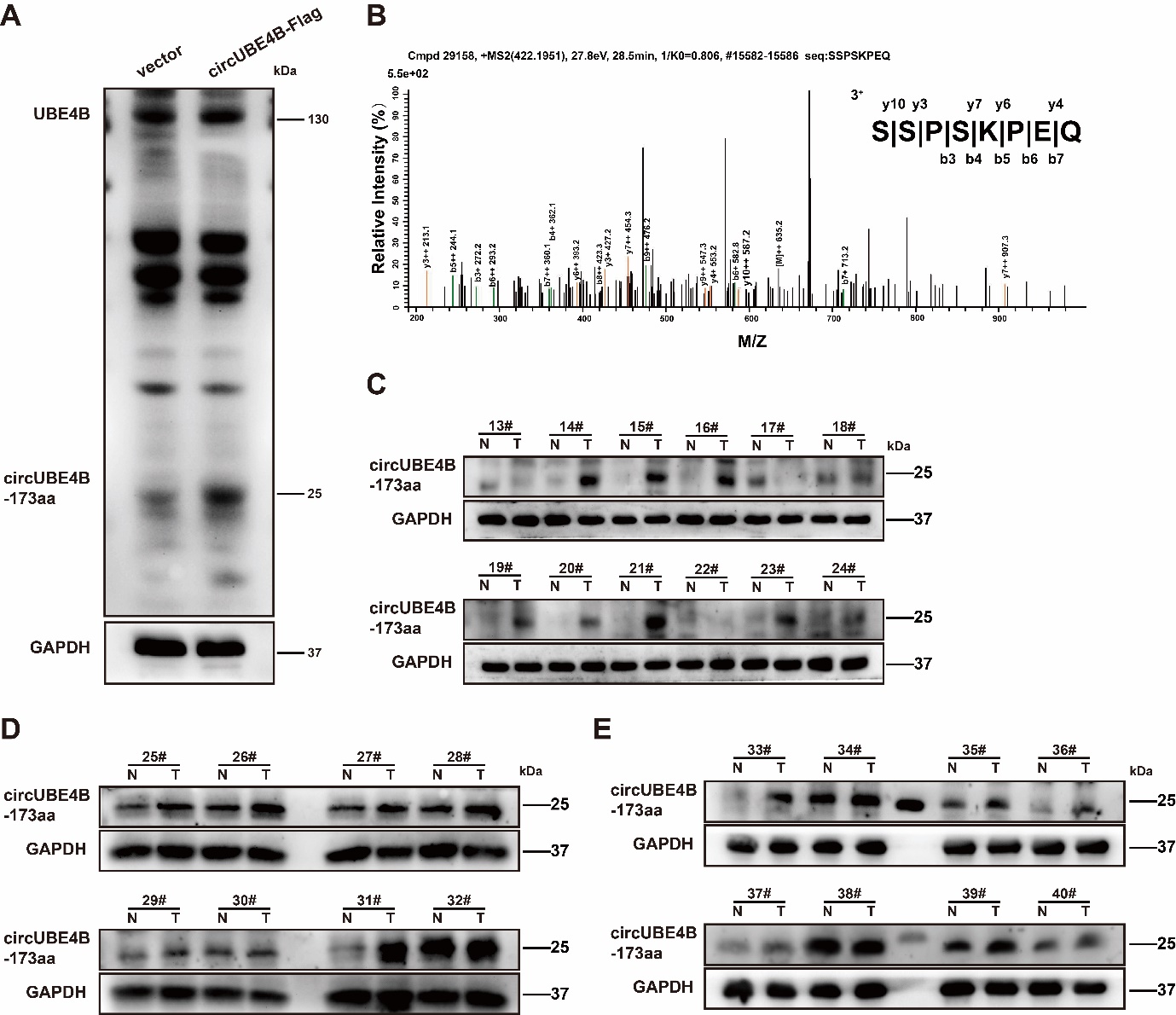
*

A. UBE4B and circUBE4B-173aa expression were detected in the ESCC cells transfected with circUBE4B-Flag plasmids.
B. LC-MS analysis was performed to identify specific peptide sequences (SSPSKPEQ) of circUBE4B-173aa.

C-E. circUBE4B-173aa expression were detected by Western Blot in ESCC tissues and paired adjacent normal tissues.

##
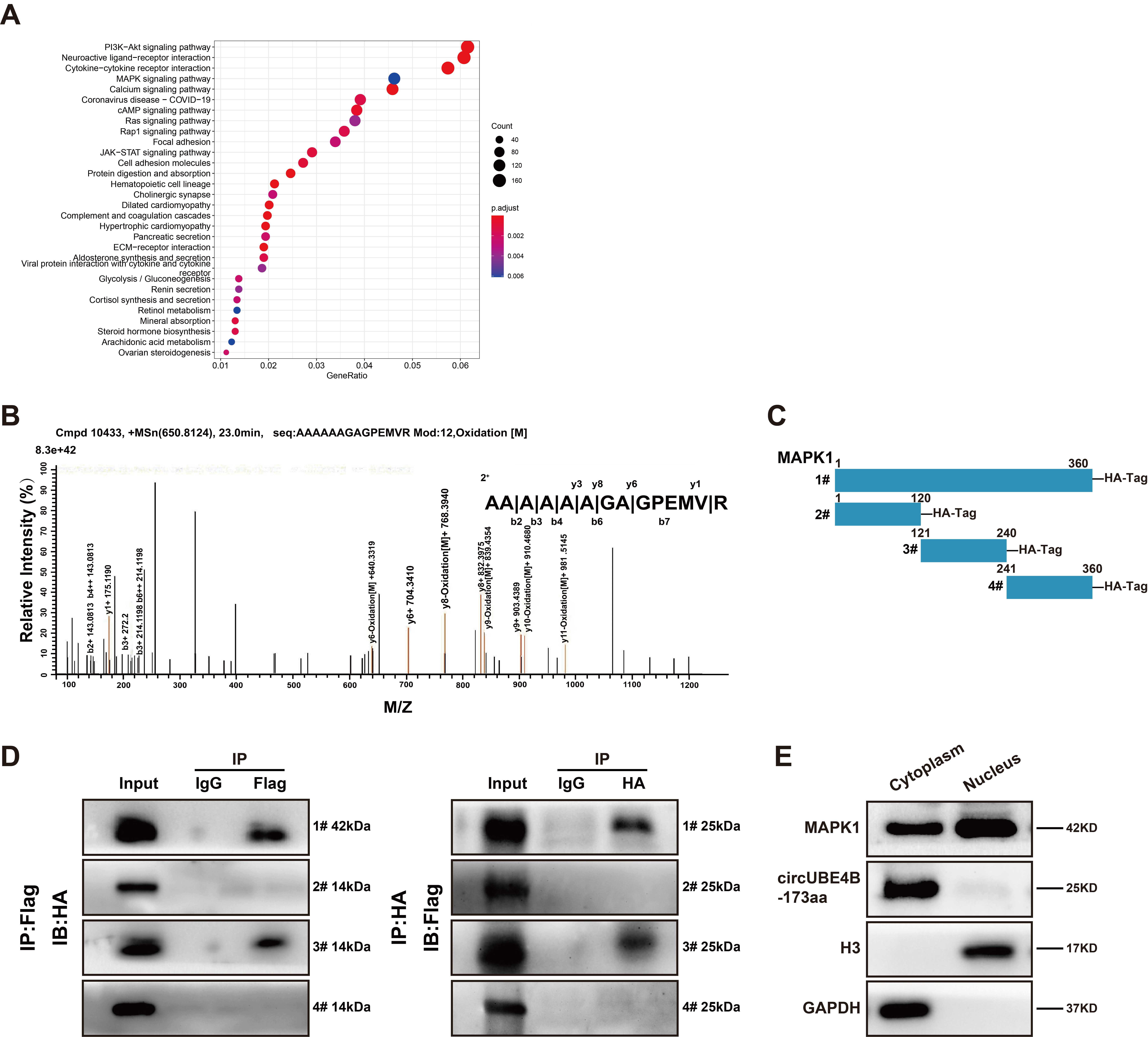
Supplemental Figure S2 *related to Figure 5*

A. KEGG signaling pathway enrichment analysis demonstrated significantly altered signaling pathways.
**B.** LC-MS analysis was performed to identify the sequences (AAAAAAGAGPEMVR) of MAPK1.

C. Schematic illustration showing that the MAPK1 protein was divided into three fragments and labeled with HA tags.

D. The direct mutual interactions of circUBE4B-173aa with different domains of HA-tagged MAPK1 were tested using immunoprecipitation.

E. Subcellular fractionation assays identified the cytoplasmic cellular localization of MAPK1 and circUBE4B-173aa in the indicated cells transfected with circUBE4B-Flag plasmids. GAPDH and H3 were used as positive controls in the cytoplasm and nucleus, respectively.
