# Supplementary material for: A novel protein encoded by circUBE4B promotes progression of esophageal squamous cell carcinoma by augmenting MAPK/ERK signaling
Source: Cell Death Dis. 2023 Jun 1;14(6):346. doi: 10.1038/s41419-023-05865-2 (PMC10235080; doi:10.1038/s41419-023-05865-2)

Fig. 3 F

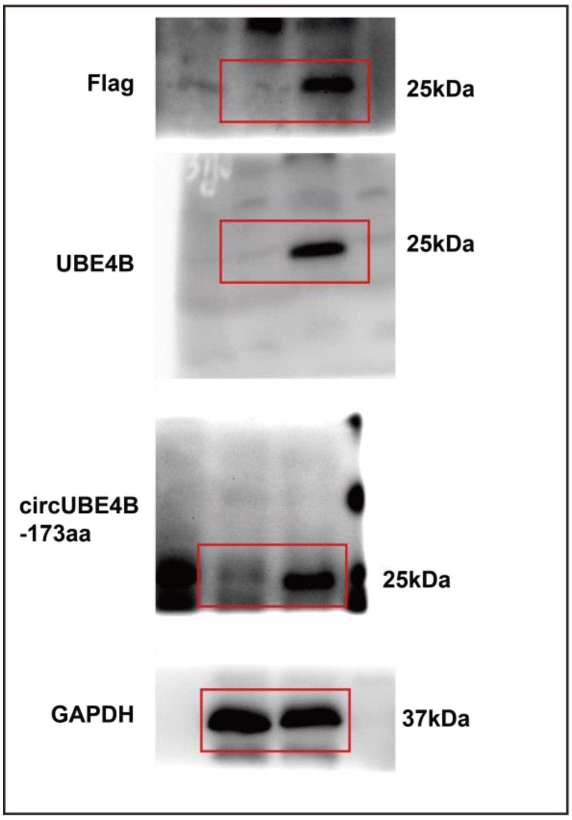

Flag
25kDa
UBE4B
25kDa
circUBE4B
-173aa
25kDa
GAPDH
37kDa

Fig. 3 I

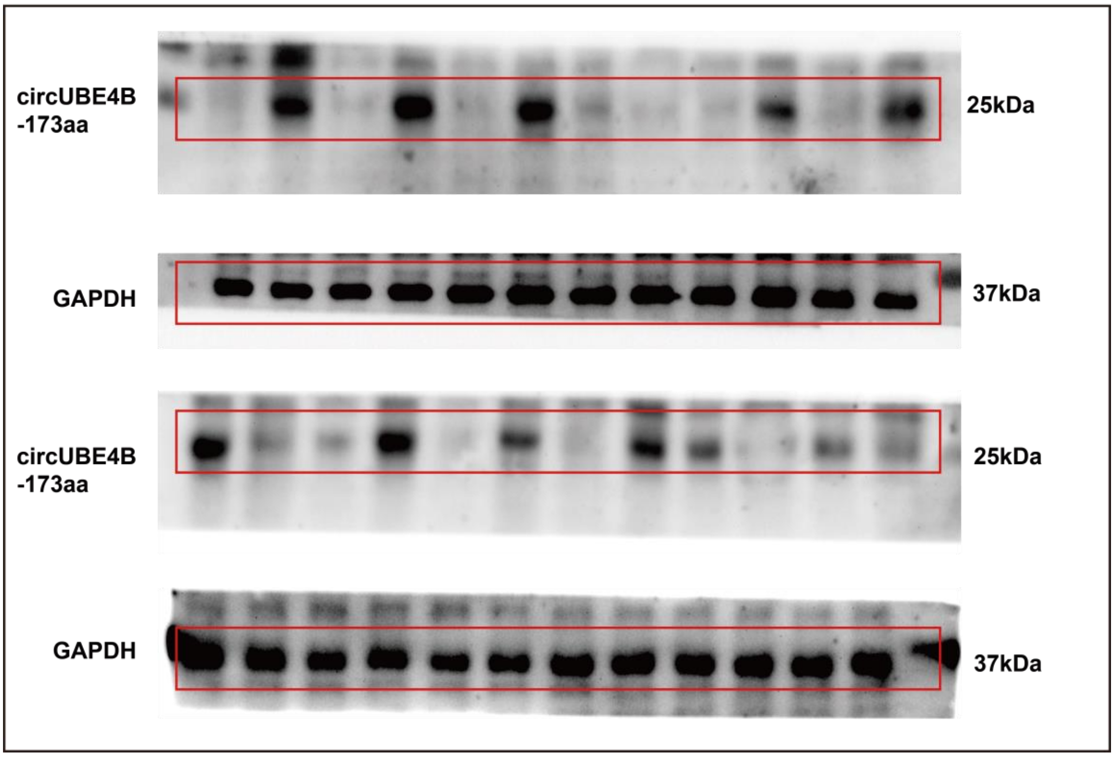

circUBE4B
-173aa
25kDa
GAPDH
37kDa
circUBE4B
-173aa
25kDa
GAPDH
37kDa

Fig. 4 C

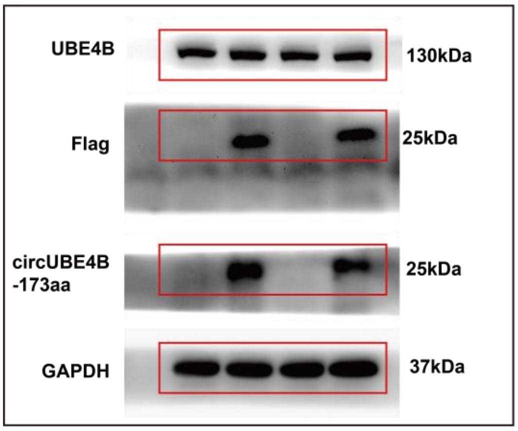

Fig. 5 E

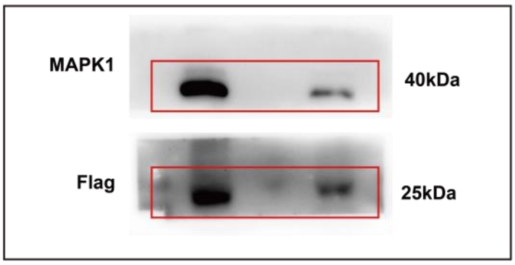

Fig. 5G

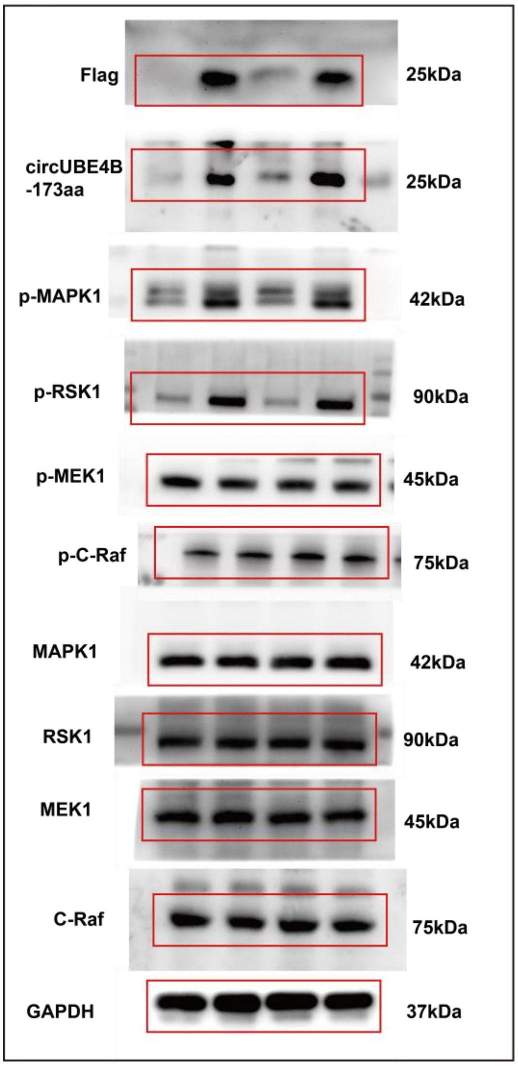

Fig. 5H

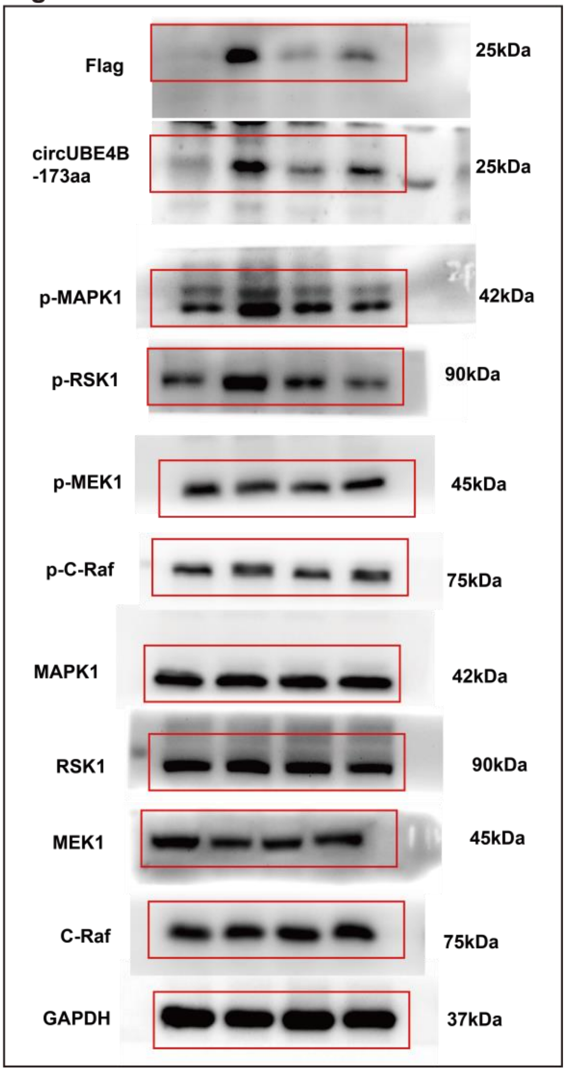

Fig. S1A

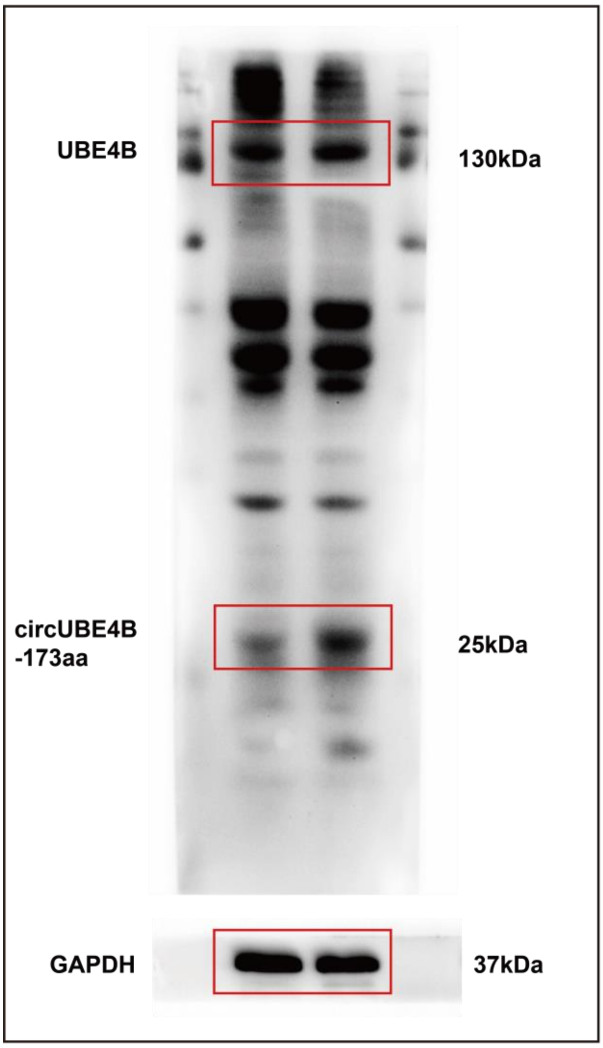

Fig. S1C

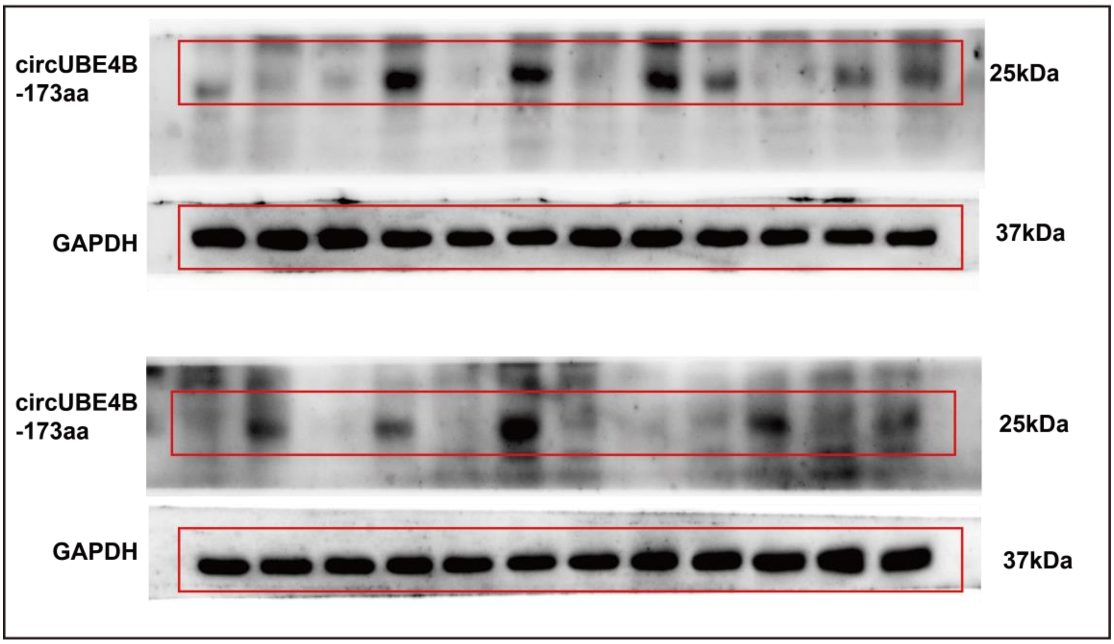

Fig. S1D

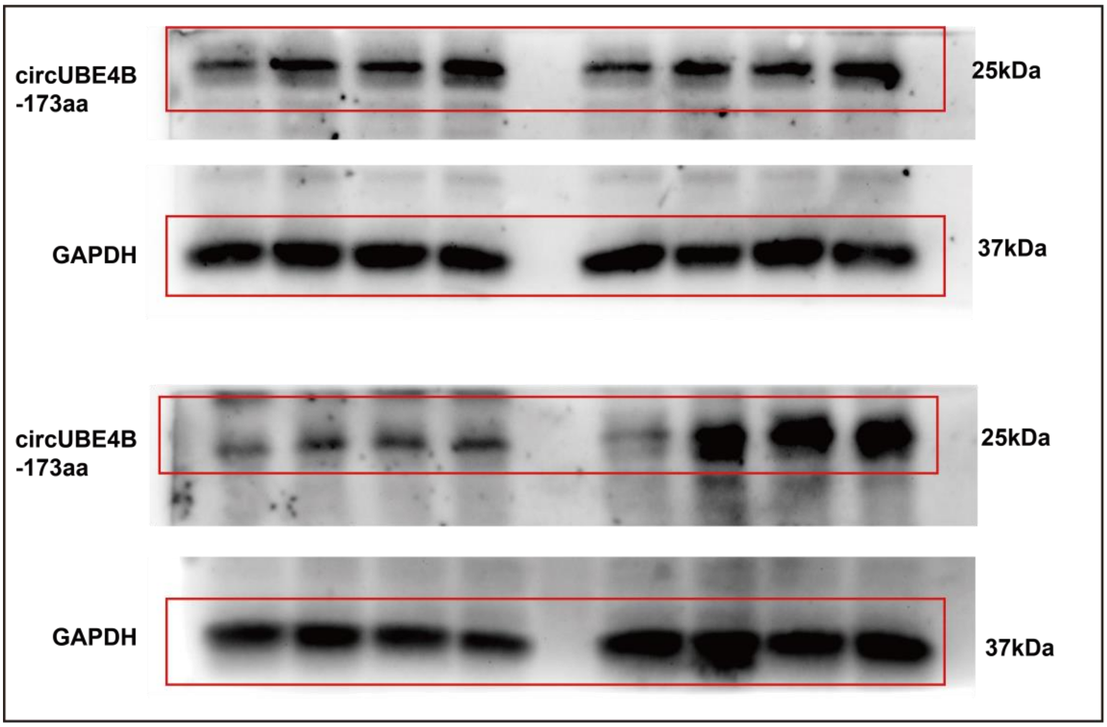

Fig. S1E

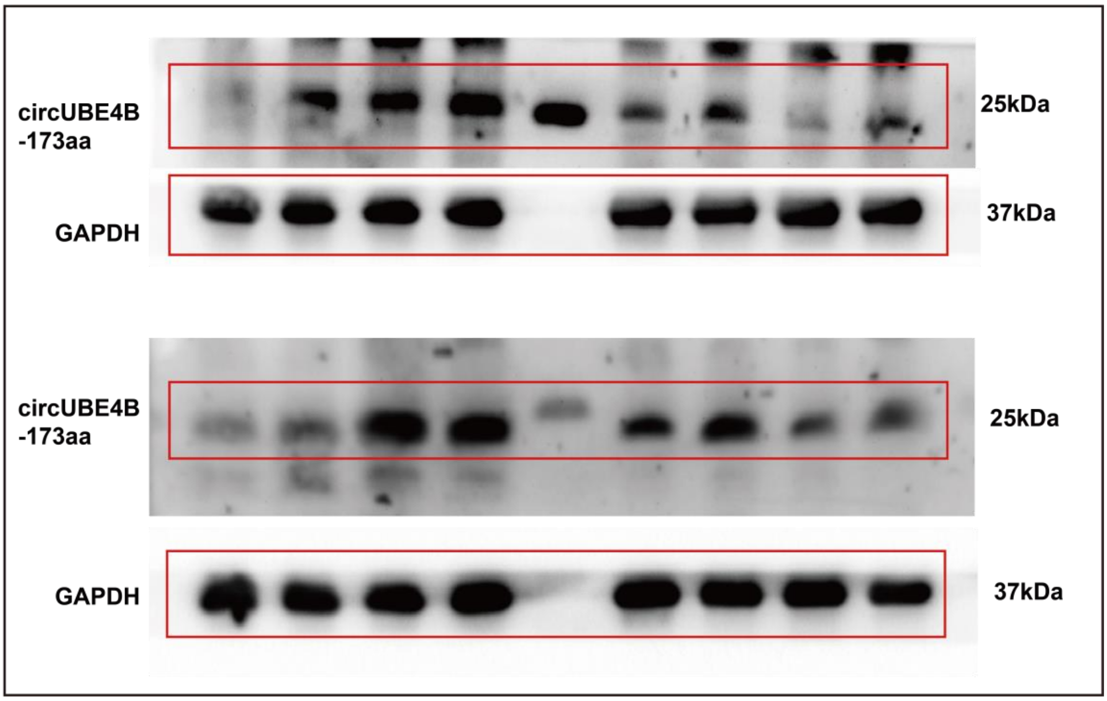

Fig. S2D

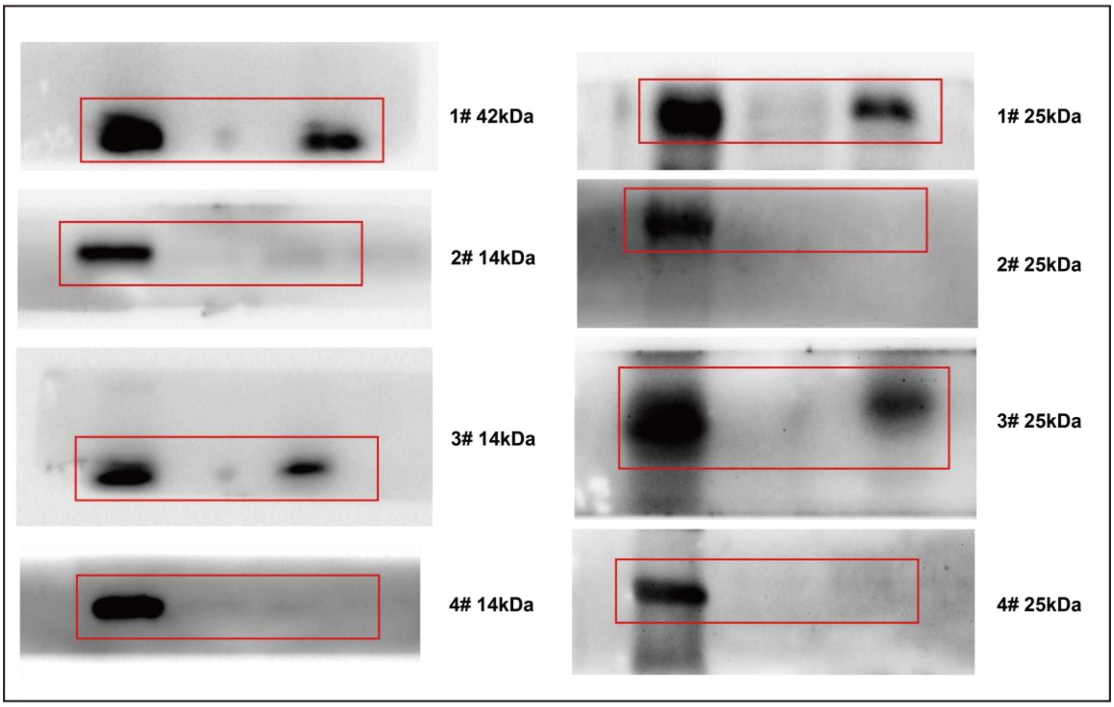

Fig. S2E

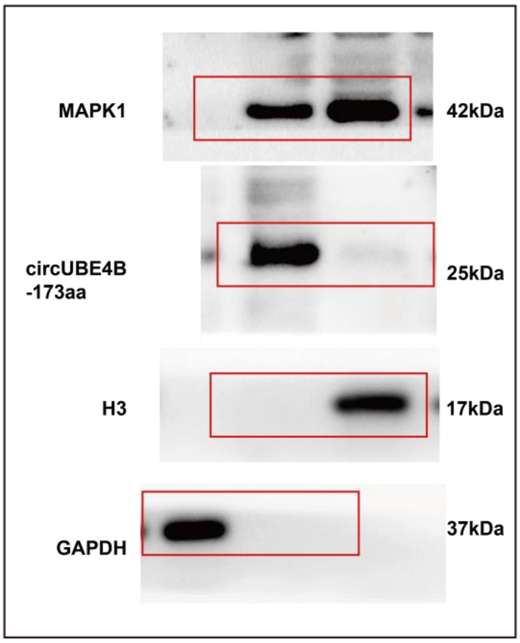

Supplement: Supplementary file 4 — Original WB [file 41419_2023_5865_MOESM4_ESM.pdf]
